# Interaction of cardiac leiomodin with the native cardiac thin filament

**DOI:** 10.1371/journal.pbio.3003027

**Published:** 2025-01-30

**Authors:** Madison Little, Cristina M. Risi, Tania M. Larrinaga, Mason D. Summers, Tyler Nguyen, Garry E. Smith, Jennifer Atherton, Carol C. Gregorio, Alla S. Kostyukova, Vitold E. Galkin

**Affiliations:** 1 Voiland School of Chemical Engineering and Bioengineering, Washington State University, Pullman, Washington, United States of America; 2 Department of Biomedical and Translational Sciences, Macon & Joan Brock Virginia Health Sciences at Old Dominion University, Norfolk, Virginia, United States of America; 3 Department of Cellular and Molecular Medicine and Sarver Molecular Cardiovascular Research Program, University of Arizona, Tucson, Arizona, United States of America; 4 Department of Medicine and Cardiovascular Research Institute, Icahn School of Medicine at Mount Sinai, New York, New York, United States of America; King’s College London, UNITED KINGDOM OF GREAT BRITAIN AND NORTHERN IRELAND

## Abstract

Every heartbeat depends on cyclical contraction-relaxation produced by the interactions between myosin-containing thick and actin-based thin filaments (TFs) arranged into a crystalline-like lattice in the cardiac sarcomere. Therefore, the maintenance of thin filament length is crucial for myocardium function. The thin filament is comprised of an actin backbone, the regulatory troponin complex and tropomyosin that controls interactions between thick and thin filaments. Thin filament length is controlled by the tropomodulin family of proteins; tropomodulin caps pointed ends of thin filaments, and leiomodin (Lmod) promotes elongation of thin filaments by a “leaky-cap” mechanism. The broader distribution of Lmod on the thin filament implied to the possibility of its interaction with the sides of thin filaments. Here, we use biochemical and structural approaches to show that cardiac Lmod (Lmod2) binds to a specific region on the native cardiac thin filament in a Ca^2+^-dependent manner. We demonstrate that Lmod2’s unique C-terminal extension is required for binding to the thin filament actin backbone and suggest that interactions with the troponin complex assist Lmod2’s localization on the surface of thin filaments. We propose that Lmod2 regulates the length of cardiac thin filaments in a working myocardium by protecting newly formed thin filament units during systole and promoting actin polymerization at thin filament pointed ends during diastole.

## Abstract 2

Maintenance of the length of actin-based thin filaments in the human heart is crucial for myocardium function, but the mechanisms underlying this process remain unclear. Here, the authors show that cardiac leiomodin regulates the length of thin filaments by protecting newly formed filament units during systole and promoting actin polymerisation during diastole.

## Introduction

**Fig 1:**
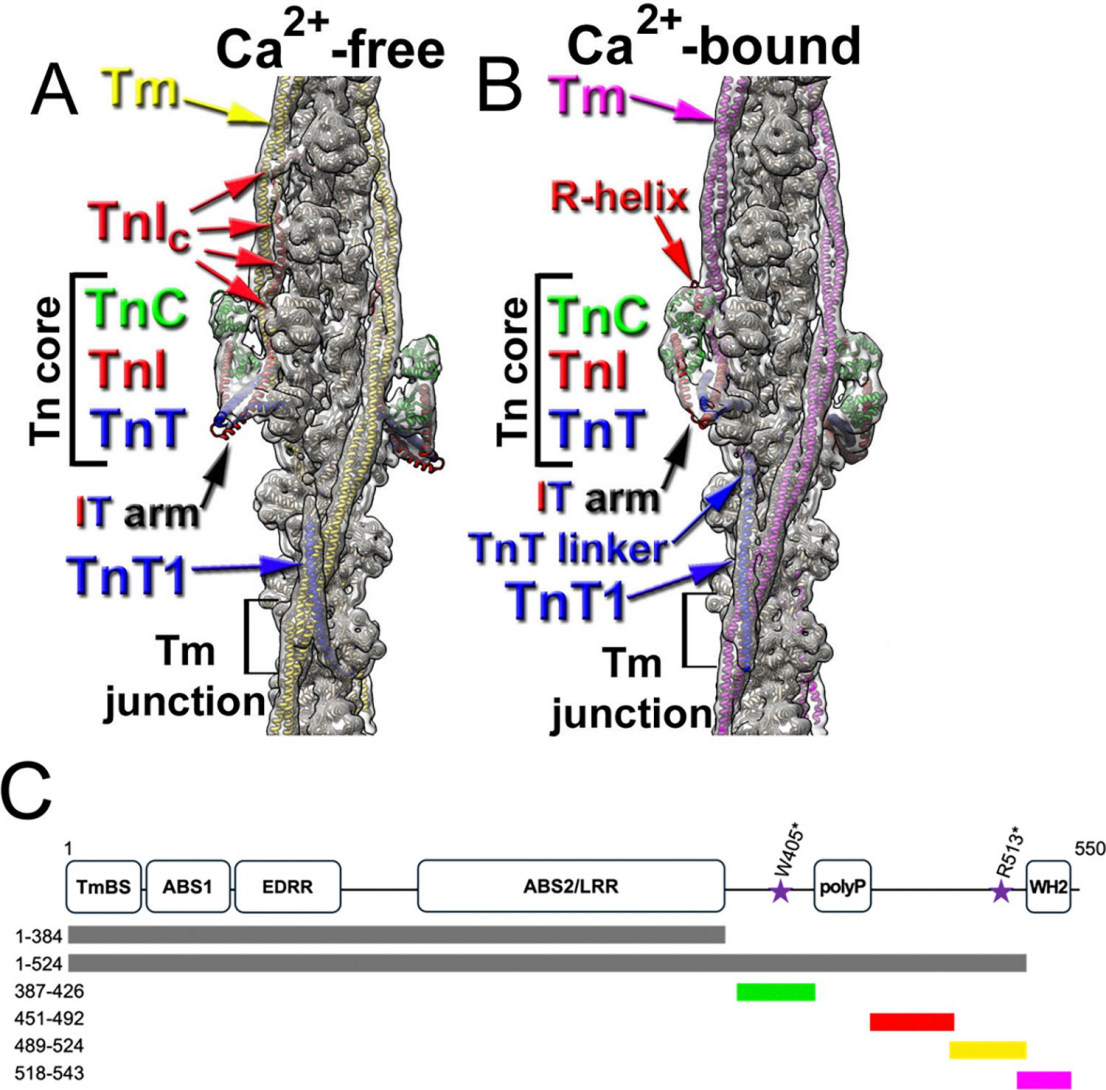
Composition of the cardiac TF and schematic representation of Lmod2 domain layout and its fragments used in the manuscript. (A, B) The models of the cTF in Ca^2+^-free *relaxed* (A) and Ca^2+^-bound *activated* (B) states [4,5] (colored ribbons) are docked into the overall 3D reconstructions (gray transparent surfaces) of the cardiac TF in either Ca^2+^-free (A) or Ca^2+^-bound (B) conformations. The TF models consist of actin (tan ribbons), Tn core (large square brackets) comprised of TnC, TnI, and TnT (green, red, and blue ribbons, respectively), and Tm molecules shown as yellow ribbons for Ca^2+^-free (A) and magenta ribbons for Ca^2+^-bound (B) states. TnT is a part of both Tn core (large brackets) and junction region (small brackets). TnT1 position in the junction region is marked with blue arrows. The portion of the TnT that connects Tn core with the TnT1 region that is visible only in the *activated* cTF (B) is portrayed as TnT linker (blue arrow). At low Ca^2+^ TnI C-terminus spans up from the Tn core over 3 actin subunits (A, red arrows), while at high Ca^2+^ only TnI C-terminal “relay helix” (B, R-helix) is visualized bound to the Tn core (B, red arrow). (C) Lmod2 contains the following regions: a tropomyosin-binding site (TmBS), ABS1, Glu(E)/Asp(D)-Rich Region (EDRR), LRR/ABS2, poly-proline domain (polyP), and a WH2 domain. Truncated Lmods (Lmod2[1–384] and Lmod2[1–524]) are shown as gray lines. Lmod2 fragments are as follows: Lmod2[387–426] (green), Lmod2[451–492] (red), Lmod2[489–524] (yellow), and Lmod2[518–543] (magenta). The stars show the positions of cardiomyopathy associated nonsense mutations W405* and R513*. ABS1, actin-binding site 1; ABS2, actin-binding site 2; LRR, leucine-rich repeat; WH2, Wiskott–Aldrich homology 2.

Striated muscle contraction is crucial for life. Striated muscle tissue is made of repeating contractile units called sarcomeres. The main functional elements of the sarcomere are myosin-based thick filaments and actin-based thin filaments (TFs) that interact to produce contractile force [1,2]. The activation and relaxation of cardiac muscle is tightly regulated by sarcomeric proteins and free Ca^2+^ concentration; Ca^2+^ activates the TFs. TFs are built on the filamentous actin (F-actin) backbone (Fig 1, tan ribbons), which anchors tropomyosin (Tm) (Fig 1, yellow (A) and magenta (B) ribbons) and the troponin (Tn) complex comprised of Ca^2+^ binding TnC (Fig 1, green ribbons), tropomyosin interacting TnT (Fig 1, blue ribbons), and inhibitory TnI (Fig 1, red ribbons). TnC binds Ca^2+^ which leads to dissociation of the TnI C-terminus (Fig 1A, red arrows) from its actin-Tm interface triggering relocation of the Tm cable from the myosin binding sites on actin promoting active cross-bridge formation [2–6]. The TnT N-terminus, named hypervariable region (human residues 1–75), is not visible in the cryo-EM maps, and its role in TF regulation is not established. The following Tm binding part of Tn (human residues 87–150) is termed TnT1 (Fig 1, TnT1); it stabilizes the junction between the 2 overlapping Tm molecules (Fig 1, black bracket, Tm junction). The so-called linker region (human residues 151–200) that connects TnT of the Tn core with TnT1 is not visible in the cryo-EM map of the relaxed cardiac TF but forms an α-helical density between the 2 strands of the activated TF (Fig 1B, blue arrow). The TnT linker’s functional role is not well defined, but hypertrophic and dilated cardiomyopathies related to mutations in the TnT linker [7–9] point to its role in cardiac TF regulation.

In addition to Ca^2+^, the strength and dynamics of heart contraction are regulated by sarcomeric proteins. For example, myosin-binding protein C (MyBP-C) stabilizes the off state of the thick filaments [10–13] and activates TFs [14–17] in a phosphorylation-dependent manner [18,19], while the phosphorylation level of the thick filament-associated protein titin regulates mechanosensitivity of the myofilament system [20]. Nevertheless, all the aforementioned mechanisms of heart contraction rely on the crystalline-like regularity of thick and thin filaments in their length and spacing, maintained by a cohort of muscle-specific proteins [21]. In myocytes, CapZ controls TF assembly and length at their barbed ends [22], while tropomodulin (Tmod) and leiomodin (Lmod) fulfill the same task at their pointed ends. Tmod specifically prevents actin polymerization/depolymerization by forming a tight cap at the pointed end [23,24], while Lmod nucleates actin in vitro [25] and was proposed to act as a “leaky cap” at the TF pointed end, allowing pointed end elongation [26]. Therefore, Tmod and Lmod, 2 members of the tropomodulin family, have antagonistic effects on the maintenance of the length of the TF actin backbone [27]. In striated muscle, there are 2 major isoforms of Lmod, Lmod2 and Lmod3, expressed primarily in cardiac and skeletal muscle, respectively [28,29]. Knockout of Lmod2 in mice resulted in shorter TFs, and lethal dilated cardiomyopathy (DCM) [30–32]. A schematic representation of the Lmod2 domain layout is shown in Fig 1C. Lmod2 contains an N-terminal Tm-binding site (TmBS), actin-binding site 1 (ABS1), and actin-binding site 2 (ABS2) represented by the leucine-rich repeat (LRR) domain [28,29]. All of these sites are also present in Tmod. In place of the second TmBS found in Tmod, Lmod2 has a linker between its ABS1 and LRR domain that contains the glutamic and aspartic acid rich-region (EDRR) that is suggested to bind Ca^2+^ and affect the ability of Lmod2 to bind 3 actin monomers (G-actin) and form a nucleus from which polymerization can continue; so-called nucleation ability [33]. Compared to Tmod, Lmod2 has an additional C-terminal extension (approximately 20 kDa) that harbors a Wiskott–Aldrich homology domain 2 (WH2), a common G-actin binding motif [34], that serves as a third actin-binding site of Lmod2 [28,29]. Aside from the WH2 domain, little is known about the function of the C-terminal extension. Nevertheless, truncations of the Lmod2 C-terminal extension are harmful which accentuates its importance in cardiac TF maintenance. For example, human LMOD2 mutation W398* (e.g., truncation at residue 398) causes severely reduced expression of LMOD2 due primarily to nonsense mediated decay [31,35], abnormally short TFs, and decreased contractile force resulting in cardiomyopathy [31]. Expression of Lmod2 in mice with the homologous mutation W405* retained only partial functionality [31]. Another nonsense mutation in the C-terminal extension, LMOD2 R513*, resulted in severe cardiac dysfunction in humans [36].

In accordance with differences in domain organization and functional properties, Tmod and Lmod have been shown to localize differently within the sarcomere. Tmod was found to localize to narrow bands next to the M-lines, while Lmod had a broader localization with larger separation from the M-lines [37,38]. Taking into account the expected association of both proteins with the TF pointed ends residing in the M-lines, the broad localization of Lmod was suggested to be attributed to its interaction with the ends of growing TFs of different lengths [37]. Nevertheless, several groups independently demonstrated that Lmod was capable of binding to the sides of F-actin [37,39,40], providing an explanation for the broad localization of Lmod in the sarcomere.

Here, we demonstrate that Lmod2 directly interacts with the sides of native cardiac TFs via a novel actin-binding region located in the C-terminal extension between the LRR and WH2 domains. Our data strongly indicate that the TnT linker has a role in the positioning of Lmod2 on the surface of the cardiac TF. We also show that the amount of Lmod2 that binds TFs increases at high Ca^2+^ levels. Finally, we hypothesize that binding of Lmod2 to the activated cardiac TF may protect the pointed ends by moderating the formation of active cross-bridges via direct competition with the myosin heads for binding to the TF.

## Results

### Lmod2 binds to the sides of native cardiac TFs in a Ca^2+^-dependent manner

**Fig 2:**
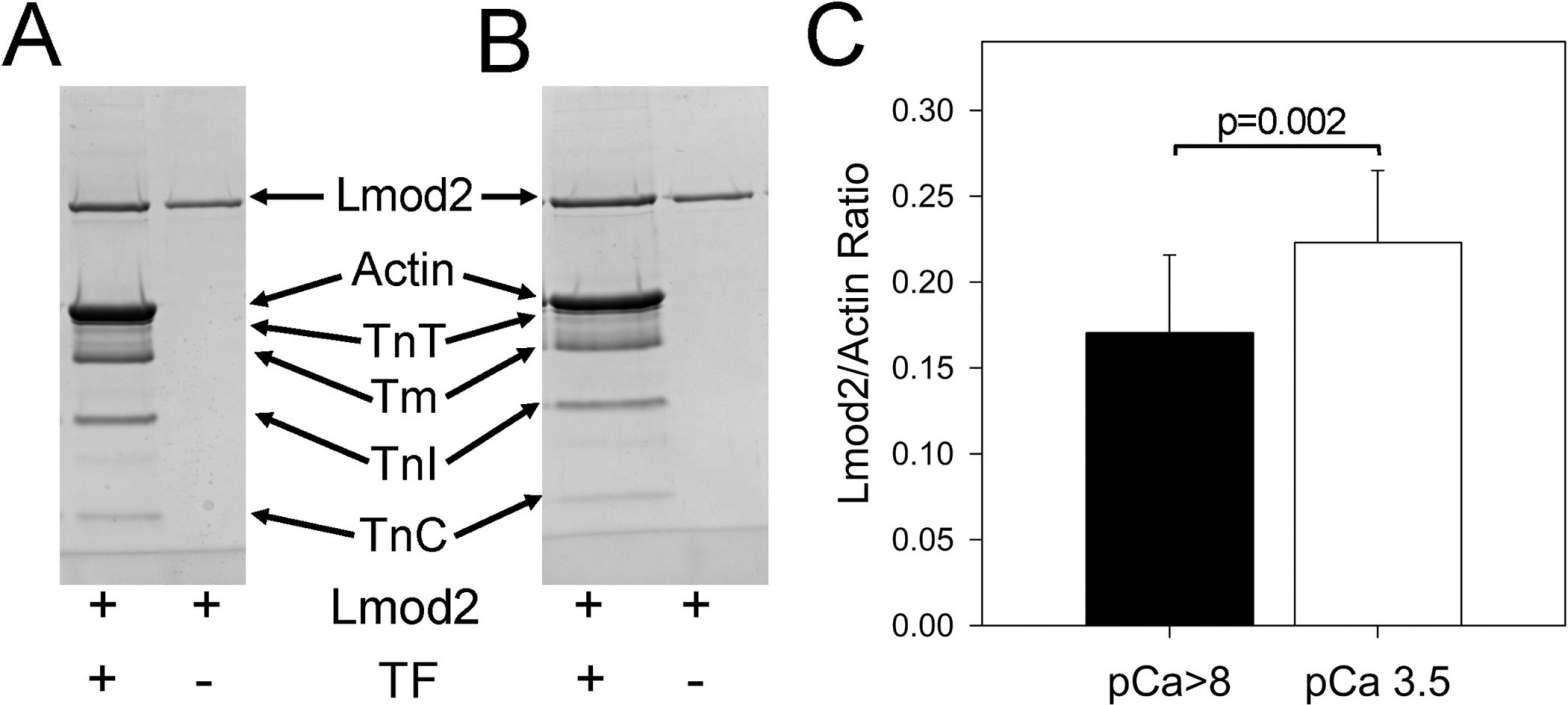
Lmod2 binds to TFs in a Ca^2+^-dependent manner. (A, B) TFs (1 μM) at either pCa >8 (A) or pCa 3.5 (B) were incubated with 1.5 μM Lmod2, centrifugated at 213,600 × g speed and pellets were analyzed by SDS-PAGE. (C) Bar graph obtained by gel densitometry of the actin and Lmod2 bands in the pellets after high speed centrifugation (213,600 × g) shows ratios of Lmod2 to actin for low (black bin) and high (white bin) Ca^2+^ levels. Lmod2 to actin density ratio in the pellets after centrifugation at 213,600 × g was calculated by subtracting the density of the Lmod2 band that pelleted in the absence of TF and dividing by the background-corrected density of actin bands. Error bars show standard deviations (*n* = 16 at pCa >8 and at pCa 3.5). The data underlying this figure can be found in either S1 Raw Images or S1 Data.

To assess whether Lmod2 may interact with the sides of the matured TFs, we performed high-speed cosedimentation of full-length Lmod2 with native cardiac TFs (cTFs) (S1 Fig). Due to the concentration-dependent intrinsic aggregation of Lmod2, we were not able to determine the stoichiometry of Lmod2 binding and its affinity for the cardiac TF in this experiment. Therefore, we repeated the cosedimentation experiment using an Lmod2 concentration of 1.5 μm to avoid excessive aggregation (Fig 2). Since the structure of cardiac TFs in the Ca^2+^-free (*relaxed*) and Ca^2+^-bound (*activated*) states is very different [4,5], we performed cosedimentation assays at low (pCa >8) and high (pCa 3.5) Ca^2+^ levels. To account for Lmod2 aggregation, the band densities corresponding to Lmod2 pelleted in the absence of TFs were subtracted from the band densities of Lmod2 pelleted in the presence of TFs. Interestingly, the cosedimentation assay revealed that Lmod2 binds more readily to the cardiac TFs at high Ca^2+^ levels.

**Fig 3:**
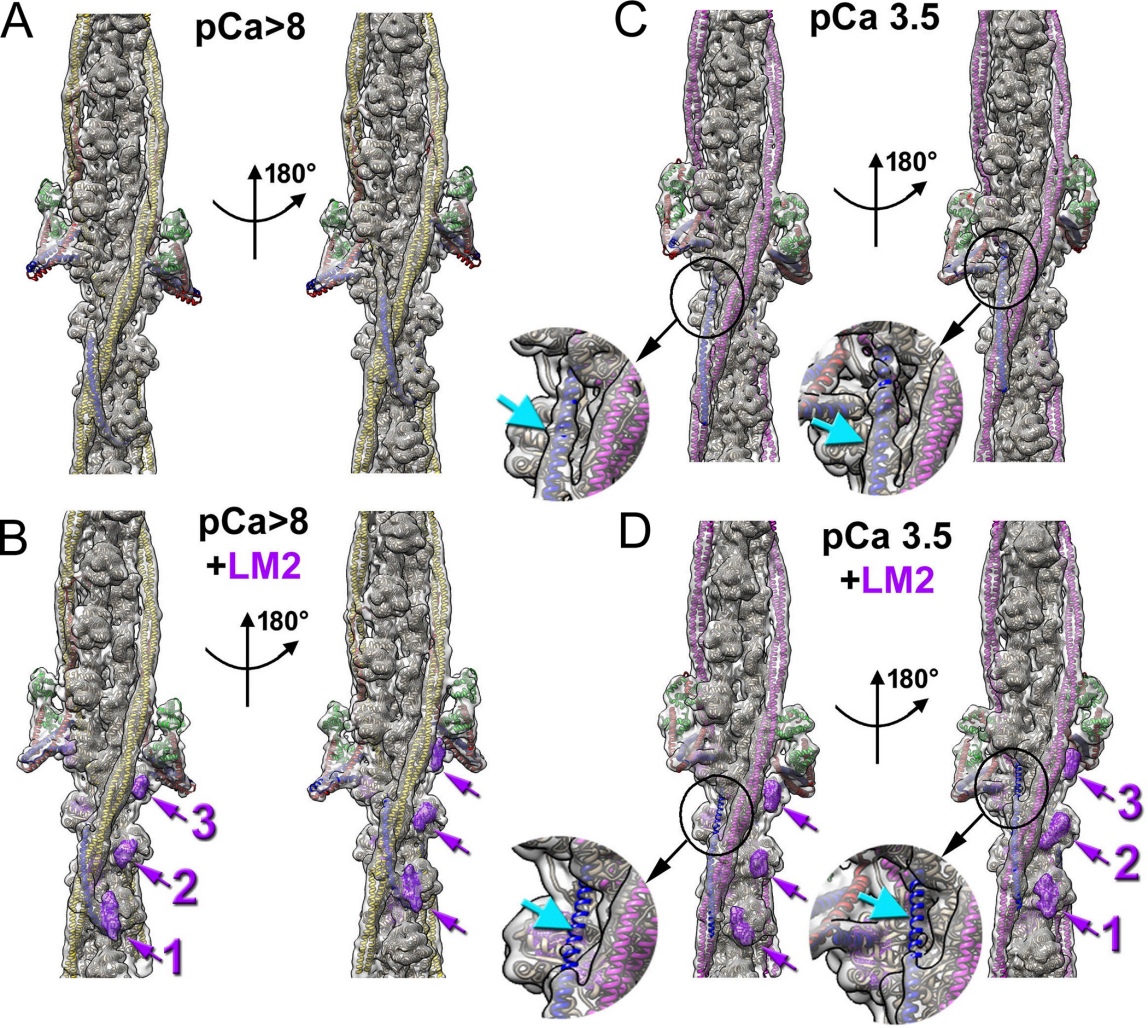
Localization of Lmod2 on the surface of the cardiac TF (cTF) at low and high Ca^2+^ levels. (A, B) 3D reconstructions (gray transparent surfaces) of the cTF in Ca^2+^-free state alone (A) or decorated with Lmod2 (B) portrayed in 2 views related by 180° azimuthal rotation. Components of the cTF are shown as colored ribbons using the same color code as in (Fig 1A). Three additional densities that represent Lmod2 bound to the cTF actin backbone are shown in (B) as a purple mesh and marked with 3 numbered purple arrows. (C, D) 3D reconstructions (gray transparent surfaces) of the Ca^2+^-bound cTF alone (C) or decorated with Lmod2 (D) depicted in 2 views related by 180° azimuthal rotation. Components of the cTF are shown as colored ribbons using the same color code as in (Fig 1B). The presence of the TnT-linker density on both strands of the Ca^2+^-bound Lmod2-free cTF is marked with cyan arrows and magnified in the insertion in (C). Three additional densities, which represent Lmod2 bound to the Ca^2+^-activated cTF actin backbone, are shown in (D) as a purple mesh and marked with 3 numbered purple arrows. Note that the TnT-linker density on both strands of the Ca^2+^-bound Lmod2-decorated cTF is absent (D, inserts and cyan arrows). Cryo-EM maps for *relaxed* and *activated* cTFs decorated with Lmod2 can be downloaded from the Electron Microscopy Data Bank (www.ebi.ac.uk/pdbe/emdb) using accession codes EMD-47712 and EMD-47714, respectively.

Next, we used cryo-EM to determine which TF components (e.g., actin, Tm, or Tn) are involved in interactions with Lmod2. Since the cosedimentation assay demonstrated Ca^2+^ dependence of Lmod2’s interaction with TFs, we generated 3D reconstructions of native cardiac TFs decorated with full-length Lmod2 at low (pCa >8) and high (pCa 3.5) Ca^2+^ levels (Fig 3). We used a previously described approach to single particle 3D reconstruction of TFs [5,6] to reveal the structure of the cardiac TF decorated with full-length Lmod2 (detailed in the SI Materials and Methods section and S2 and S3 Figs). For comparison purposes, we used naked TF maps from previously published work [6] filtered to 9 Å resolution (Fig 3A and 3C). The overall resolution of the 3D maps for the *relaxed* and *activated* TFs decorated with Lmod2 was 6.2 Å (S2F Fig) and 6.1 Å (S3F Fig), respectively. As anticipated, the resolution within the maps was not uniform and ranged from ~4.5 Å in the TF actin backbone to ~10.5 Å at the end of the IT arm tip (S2G and S3G Figs). Meanwhile, the resolution of the Lmod2 density on the actin surface and Tm density was resolved to ~9 Å. For this reason, the maps were low-pass filtered to 9 Å and are shown in Fig 3B and 3D. The only difference we found between the 3D reconstructions of undecorated (Fig 3A) and Lmod2-decorated (Fig 3B) TFs in the *relaxed* state was the addition of 3 densities spanning from the Tm junction region towards the Tn core (Fig 3B, purple mesh depicted with purple arrows and numbers). The overall structure of the *relaxed* TF was not perturbed by the bound Lmod2. In contrast, when TFs were decorated with Lmod2 in the *activated* state, an alteration in their structure was detected in the TnT1 linker region. The TnT-linker density was clearly present in the absence of Lmod2 (Fig 3C insert, cyan arrow), while it was completely missing in the Lmod2-decorated TFs (Fig 3D insert, cyan arrow). Despite this alteration of the activated TF, the structure of the rest of the cTF (at reported resolution) was not altered. The Lmod2 additional densities in the *relaxed* and *activated* TFs (Fig 3B and 3D, purple mesh with purple arrows 1–3) were very similar. The Lmod2 density on the actin protomer that harbored the Tm junction and TnT1 was the largest (Fig 3B and 3D, purple arrow 1), while the densities on the actins 2 and 3 were of comparable sizes but smaller than the first density (Fig 3B and 3D, purple arrows 2 and 3).

### The C-terminal extension of Lmod2 is required for binding to TFs

Lmod2 has 2 actin binding sites in its N-terminal and central part (Fig 1C, ABS1 and ABS2/LRR) and ABS3 in its C-terminus (Fig 1C, WH2). Since the WH2 domain is known to bind to G-actin [34] and based on the fact that the size and localization of the Lmod2 densities bound to the TF (Fig 3B and 3D) were not consistent with the LRR domain size and localization when in complex with G-actin [41], we hypothesized that the region between the LRR domain and WH2 domain could be involved in the interaction with the TF. To test this hypothesis, we used 2 Lmod2 fragments (depicted in Figs 1C and S1A): Lmod2[1–384] that was missing the whole C-terminal extension and Lmod2[1–524] that was missing the WH2 domain.

**Fig 4:**
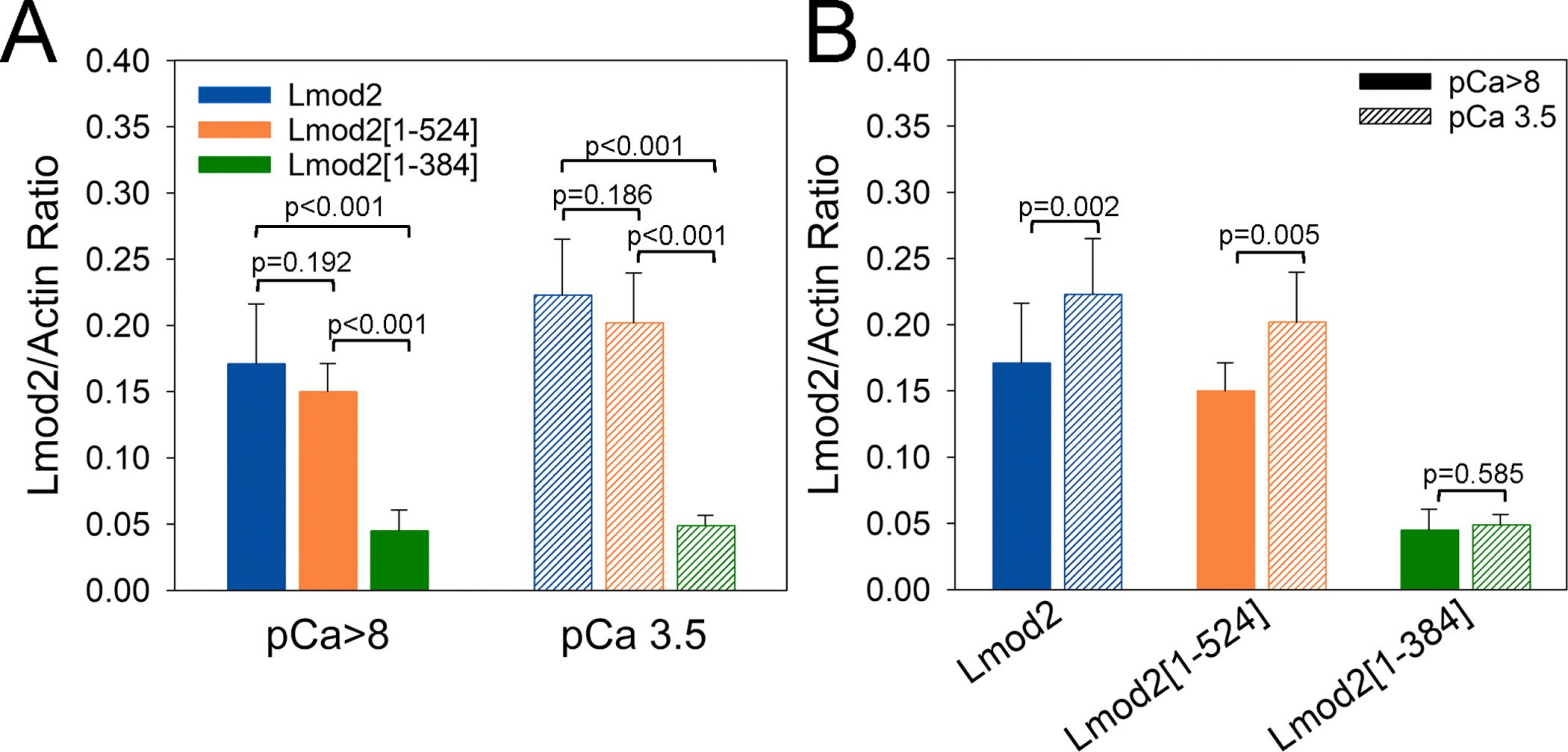
The C-terminal part of Lmod2 is required for its interaction with thin filaments. Approximately 1 μM TF was incubated with 1.5 μM full-length Lmod2, Lmod2[1–524], or Lmod2[1–384] at either pCa >8 or pCa 3.5, and pelleted at 213,600 × g. Bar graphs obtained by gel densitometry of the Lmod2 and actin bands in the pellets show ratios of Lmod2 to actin for full-length Lmod2 (blue), Lmod2[1–524] (orange), and Lmod2[1–384] (green) at pCa >8 (solid bars) and pCa 3.5 (striped bars). The Lmod2 to actin density ratios were calculated by subtracting the density of the Lmod2 that pelleted in the absence of TF and dividing by the background-corrected density of actin bands. (A) Statistical analysis of differences in binding of the Lmod2 constructs to TF. (B) Statistical analysis of differences in binding of the Lmod2 constructs to TF at pCa >8 (filled bars) and pCa 3.5 (striped bars). Error bars show standard deviations (full-length Lmod2: *n* = 16 (pCa >8 and pCa 3.5), Lmod2[1–524]: *n* = 8 (pCa >8 and pCa 3.5), and Lmod2[1–384]: *n* = 8 (pCa >8) and *n* = 7 (pCa 3.5). The data underlying this figure can be found in S1 Data.

The truncated Lmod2 fragments were compared with full-length Lmod2 at varying concentrations in cosedimentation assays with TFs at high Ca^2+^ level (S1B and S1C Fig). Lmod2[1–384] binding to TFs was insignificant (S1B and S1C Fig), while Lmod2[1–524] cosedimented with the cardiac TFs in a concentration-dependent manner (S1B Fig). Both Lmod2[1–524] and Lmod2[1–384] also aggregated at high concentrations though to a lesser extent than full-length Lmod2. The cosedimentation was repeated using 1.5 μM of Lmod2[1–524] or Lmod2[1–384] at either low or high Ca^2+^ levels, and the band densities of truncated Lmod2 pelleted with TF were corrected by subtracting the band densities of truncated Lmod2 pelleted in the absence of TFs (Figs 4 and S1B). While truncation of the WH2 domain consistently reduced the level of Lmod2 interaction with TFs (Figs 4A, orange bars and S1B), this reduction was not statistically significant at both high and low Ca^2+^ levels. This suggests that although the WH2 domain may contribute to the interaction with F-actin, it is not the major determinant for Lmod2 binding to sides of TFs. The removal of the entire Lmod2 C-terminal extension made binding to TFs negligible (Fig 4A, green bars) suggesting the pivotal role of the C-terminal extension in the Lmod2 interaction with TFs and the existence of additional actin-binding regions between the LRR and WH2 domains.

We compared the effect of Ca^2+^ levels on TF binding to Lmod2[1–524] and Lmod2[1–384] with that of the full-length Lmod2 (Fig 4B). Similar to full-length Lmod2, binding of Lmod2[1–524] to TFs was Ca^2+^ dependent (Fig 4B).

To understand the correlation between the nucleation and side-binding abilities of Lmod2, the ability of the truncated Lmod2 fragments to nucleate actin was tested and compared with that of full-length Lmod2 (S4A and S4B Fig). Both Lmod2[1–524] and Lmod2[1–384] were still able to nucleate although to a smaller extent than full-length Lmod2 with the most drastic decrease in nucleation ability when the entire C-terminal extension was removed (~26% and ~72% less at 400 μM, respectively). This result indicates that not all of the actin-binding sites within Lmod2 that participate in nucleation are involved in binding TF sides.

### Localization of actin-binding sites in the Lmod2 C-terminal extension

**Fig 5:**
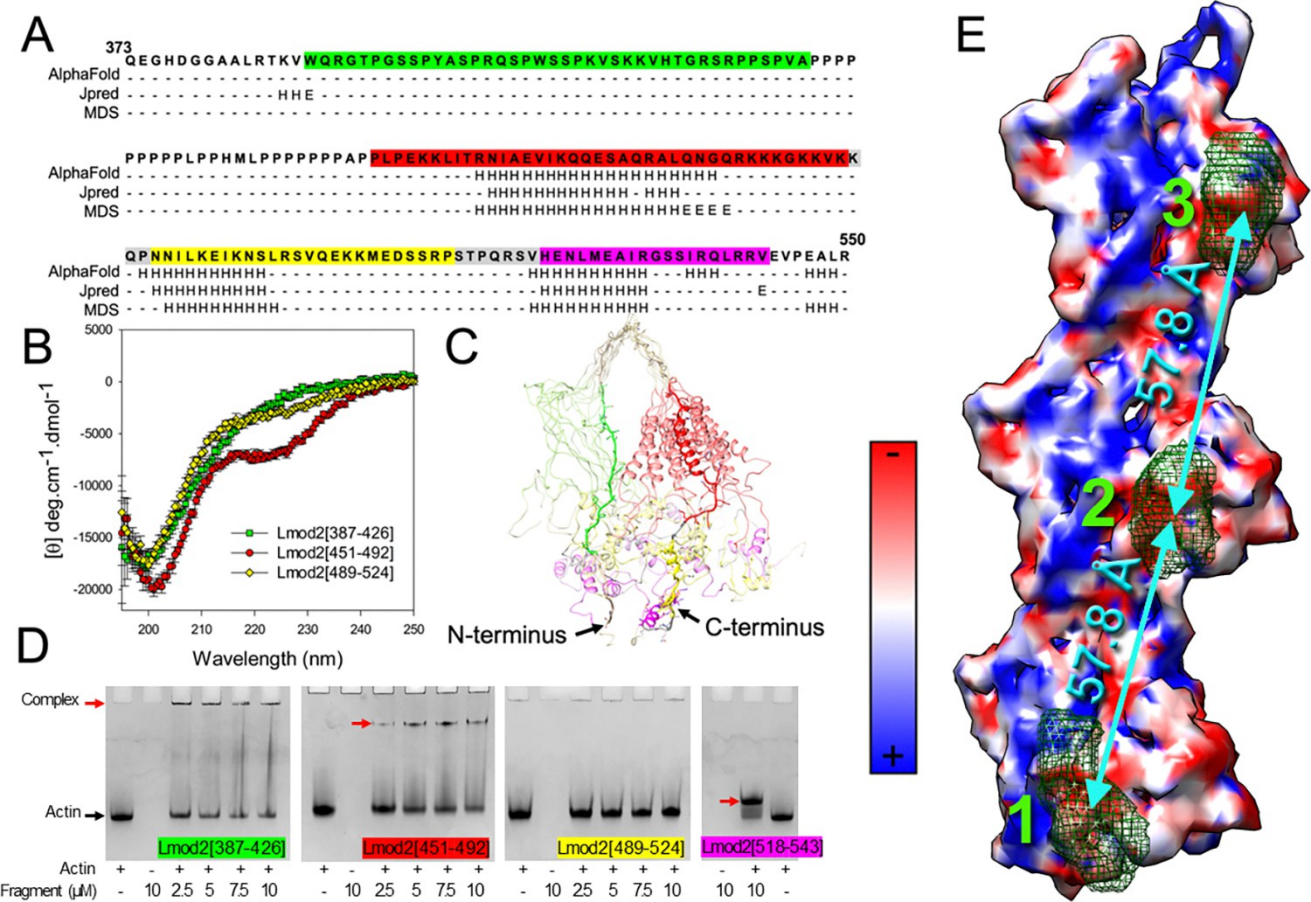
Secondary structure analysis of Lmod2 C-terminal fragments and localization of regions that bind to G-actin. (A) Alignment of Lmod2[373–550] sequence and secondary structure predictions obtained using AlphaFold [54], Jpred [62], and Amber [59]. The sequence is colored according to the C-terminal fragments used: Lmod2[387–426] (green), Lmod2[451–492] (red), and Lmod2[489–524] (yellow). The C-terminal WH2 domain of Lmod2[518–543] is colored in magenta. Overlapping regions of fragments are shown in gray. α-helical regions are indicated by H and β-strands are indicated by E. (B) CD spectra of the aforementioned Lmod2 fragments measured at 20°C in 10 mM sodium phosphate pH 7.0, containing 100 mM NaCl. The data underlying this figure can be found in S1 Data. (C) The average structure of Lmod2[373–550] obtained by MDS using 40,000–82,000 ps simulation times (show in bright color), overlaid on an ensemble of transient structures from the same period (shown in dim color). (D) Native PAGE showing complex formation between G-actin and the C-terminal Lmod2 fragments. Lmod2[518–543] containing the WH2 domain was used as a positive control for binding. Red arrows show the positions of bands representing the complexes, and the black arrow shows the position of the actin band. The data underlying this figure can be found in S1 Raw Images. (E) The Lmod2 density from the cryo-EM map shown in Fig 3D (green meshwork) is superimposed onto the electrostatic surface representation of the actin filament to show that Lmod2 binds to negatively charged exposed amino acids (colored in red). Positively charged exposed amino acids are in blue while neutral are in white. The distance between the Lmod2 densities on the 3 consecutive actin protomers is 57.8 Å (cyan arrows). CD, circular dichroism; MDS, molecular dynamics simulation.

Within the C-terminal extension, there is a region of repeated proline residues (polyP) common in actin-binding proteins and shown to bind profilin [42]. To better understand which parts of the Lmod2 C-terminal extension are involved in TF binding, we divided the C-terminal extension into 4 fragments (Figs 1C and 5A). Lmod2[387–426] (Figs 1C and 5A, marked in green) contained the residues between the LRR domain and the polyP region. The region after the polyP region was split into 3 overlapping fragments: Lmod2[451–492], Lmod2[489–524], and Lmod2[518–543] with the last one containing the WH2 domain (Figs 1C and 5A, marked in red, yellow, and magenta, respectively). Overlapping regions are shown in gray (Fig 5A).

First, we compared the secondary structure characteristics of the C-terminal fragments using circular dichroism (CD) spectra (Fig 5B) with the ones predicted computationally (Fig 5A). Based on the spectra shapes, Lmod2[387–426] was mostly disordered, while both Lmod2[451–492] and Lmod2[489–524] contained α-helices, with Lmod2[451–492] having the highest α-helical content (Fig 5B). AlphaFold was used to create a predicted structure of the C-terminal extension of Lmod2 (residues 373–550) using backbone dihedral angles, and a 200-ns molecular dynamics simulation (MDS) was performed to assess secondary structure stability. Results from MDS (Fig 5C) were in agreement with the computational secondary structure predictions (Fig 5A), the CD data (Fig 5B), and crystallography data for Lmod2’s WH2 domain (PDB ID 5WFN). There were no α-helices in Lmod2[387–426], an α-helical region in Lmod2[451–492] comprised of 17 residues, and shorter α-helical regions in Lmod2[489–524] and Lmod2[518–543] (Fig 5A).

Cryo-EM established that Lmod2 binds to the front of subdomain 1 of actin (Fig 3B and 3D, purple arrows) suggesting that its TF-binding regions should bind equally well to both G- and F-actin. We tested the binding of the 4 C-terminal fragments to G-actin using native gel electrophoresis (Fig 5D). Calculated isoelectric points (pI) of Lmod2[387–426], Lmod2[451–492], Lmod2[489–524], and Lmod2[518–543] were established to be 12.02, 10.86, 10.16, and 12.0, respectively. They could not be seen in the gel because their positive charges prevented them from entering the gel. If there was binding between the Lmod2 fragments and G-actin (calculated pI 5.17), we would expect to see a reduction in the intensity of the actin band, as well as the appearance of a new band that corresponds to a complex.

As expected, Lmod2[518–543], which contains the WH2 domain, formed a complex with G-actin, as observed by the actin band almost completely disappearing and a new band appearing (Fig 5D, red arrow). We also observed a concentration-dependent decrease in the intensity of the actin band and the appearance of new bands (Fig 5D, red arrows) in the presence of Lmod2[387–426] and Lmod2[451–492] indicating the formation of complexes between those fragments and G-actin. Based on the gel densitometry analysis of the actin band at a 2:1 fragment to actin molar ratio, ~90% of the actin formed a complex with Lmod2[518–543], ~70% of the actin formed a complex with Lmod2[387–426], and ~60% of the actin formed a complex with Lmod2[451–492]. No change in the density of the actin band and no appearance of a new band were observed in the presence of Lmod2[489–524] indicating that this fragment did not form a complex with G-actin in this assay. To summarize, our data demonstrated that in addition to the WH2 domain region, there are 2 new regions in the Lmod2 C-terminal extension that can bind actin. These regions can either form independent actin-binding sites or combine to form a high-affinity actin-binding site in full-length Lmod2.

Since all actin-binding regions of the Lmod2 C-terminal extension were positively charged, we evaluated the positioning of Lmod2 density on the F-actin surface with respect to actin’s surface electrostatic potential (Fig 5E). Notably, all 3 Lmod2 densities (Fig 5E, green meshwork marked with green numbers) were located on the negatively charged surface of subdomain 1 of actin (shown in red), thus, providing structural support for the biochemical data.

At both high and low Ca^2+^ levels, Lmod2 binds to 3 consecutive actin protomers (Figs 3B, 3D, and 5E). The distance from the first density (Fig 5E, number 1) to the second one (Fig 5E, number 2) is 57.8 Å (Fig 5E, cyan arrow), while this distance to the third Lmod2 density (Fig 5E, number 3) is twice as large (115.6 Å). Based on the MDS results, the maximum end-to-end distance for the whole C-terminal extension is ~120 Å (S5A Fig). The maximum end-to-end distances for both the 24-residue polyP region, which connects the first 2 actin-binding regions in the C-terminal extension, and the 35-residue region between the second and third (the WH2 domain) actin-binding regions can reach ~70 Å (S5B and S5C Fig, respectively). By combining the distances between actin molecules with Lmod2 densities (Fig 5E) with the maximum distances obtained by MDS, we can conclude that each of the 3 Lmod2 C-terminal ABSs binds to 1 actin protomer giving rise to 3 Lmod2 densities on the surface of the cardiac TF.

## Discussion

**Fig 6:**
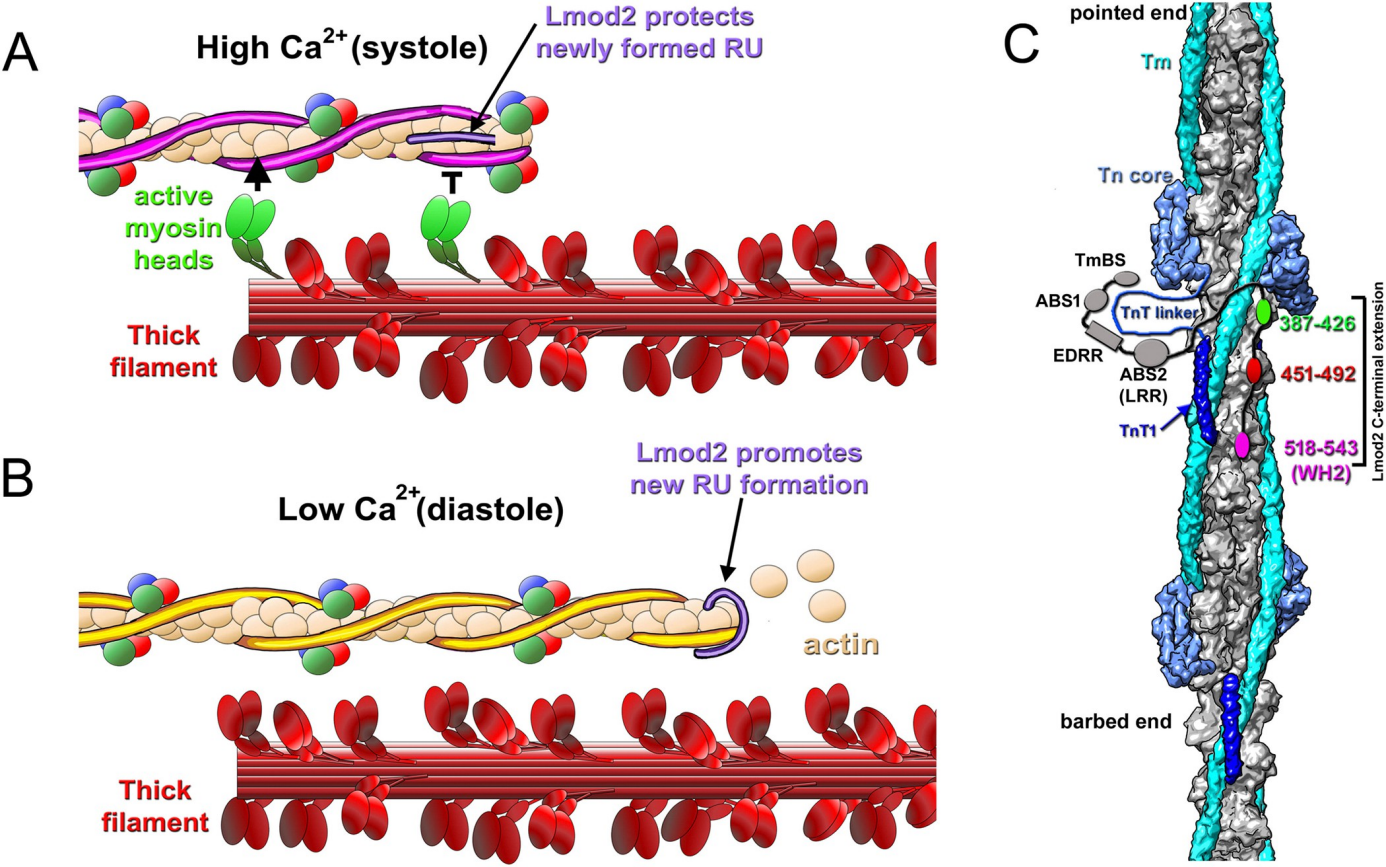
Hypothetical model of Lmod2 regulation of cardiac TF length. (A) At high Ca^2+^ levels (during systole) Lmod2 binds to the sides of TFs to protect newly formed RUs from the mechanical stress produced by active cross-bridges. (B) At low Ca^2+^ levels (during diastole) Lmod2 binds to the ends of TFs to promote their growth via a “leaky-cap” mechanism. In both (A) and (B), actin molecules are shown as tan spheres, the Tn complex is depicted as red, blue, and green spheres, while the Tm cable is shown in magenta in the *activated* state and yellow in the *relaxed* state. Thick filaments are shown in red with active myosin heads marked in green. (C) Model of Lmod2 interaction with the sides of TFs. The actin backbone is shown in gray, Tm cable in cyan, Tn core complex in light blue, and TnT1 in blue. The TnT linker is depicted as a blue line. The N-terminal elements of Lmod2, which presumably bind to the TnT linker, include the TmBS, 2 actin binding sites (ABS1 and ABS2) and Ca^2+^-binding EDRR region, which are all colored in gray. The 3 C-terminal actin binding sequences are depicted as green (residues 387–426), red (451–492), and magenta (518–543) ovals. ABS1, actin-binding site 1; ABS2, actin-binding site 2; RU, regulatory unit; TmBS, Tm-binding site.

Tmod1 and Lmod2 regulate TF length in cardiomyocytes, which is crucial to maintain sarcomere architecture. While Tmod1 expression is associated with early stages of contractile apparatus assembly [43], Lmod2 expression occurs in mature sarcomeres [27] that are actively involved in the contractile process. Hence, Lmod2 TF elongation activities may be synchronized with the Ca^2+^-regulated contraction/relaxation cycle of the cardiac muscle to protect newly formed regulatory units from mechanical force of active cross-bridges. Mechanical stress produced by myosin has been shown to depolymerize F-actin [44]. This would imply that Lmod2 may elongate cardiac TFs during the low Ca^2+^ relaxation phase (e.g., diastole) and protect newly formed pointed ends on elongated TFs during the high Ca^2+^ contraction phase (e.g., systole). Previously, we demonstrated that the actin nucleation ability of Lmod2 is significantly reduced at high Ca^2+^ levels [45]. We proposed that at high Ca^2+^ levels, Lmod2 is less active in promoting TF growth. Here, we show that high Ca^2+^ promotes Lmod2 binding to the sides of mature cardiac TFs (Figs 2 and 4). Our structural data suggests that binding of Lmod2 to TF should inhibit actomyosin interactions due to competition of Lmod2 with myosin heads (S6A Fig). The inhibition of the actomyosin interactions by Lmod2 has been shown previously with unregulated actin filaments [39]. Since Lmod2 binds to 3 preferable sites on the TF (Figs 3B, 3D, S6B, and S6C), it should inhibit only myosin binding sites at or above the junction region towards the pointed end of the filament (S6D Fig, red arrows) where TF elongation takes place. Altogether, our data lead to a novel hypothesis that Lmod2 may regulate the length of TFs in a working myocardium (Fig 6A and 6B) by protecting newly formed regulatory units during systole (Fig 6A) and promoting actin polymerization at TF pointed ends during diastole (Fig 6B). This hypothesis requires further experimental support.

In the cardiac sarcomere, Tmod1 localizes to narrow bands next to the M-lines, while Lmod2 localizes to broader bands [37,38]. To explain the Lmod2 broad localization pattern in myofibrils, 2 profoundly different mechanisms of Lmod2 interaction with TFs were proposed. The first mechanism suggested that Lmod2 interacts with ends of the growing TFs of varying lengths [37], while the second proposed mechanism suggests that the broad localization of Lmod2 is accounted for by the interaction of Lmod2 not only with the pointed ends but with the sides of the thin filaments [39]. The reported bundling of F-actin by Lmod2 is consistent with the ability of Lmod2 to bind to the sides of F-actin [40]. Here, we confirm that Lmod2 interacts with the sides of actin filaments which serve as backbones of native cardiac TFs (Figs 2 and 3).

Lmod2 binds cardiac TFs more readily at high Ca^2+^ levels (Figs 2 and 4). Nevertheless, its interaction pattern on the surface of the TF is very similar at either low or high Ca^2+^ levels (Fig 3B and 3D). These observations imply that the Ca^2+^ changes the conformation of Lmod2 so that it has a higher affinity for the TF at elevated Ca^2+^ levels while it does not change the mode of Lmod2 C-terminus binding to the TF. It is likely that Ca^2+^ binding to Lmod2’s EDRR [45], which reduces Lmod2-dependent actin nucleation, also promotes Lmod2 binding to the sides of TFs.

We show that the Lmod2 C-terminal extension is fully responsible for binding to the sides of TFs (Fig 4). This excludes ABS1 and ABS2 (LRR domain) of Lmod2 from participation in the binding to the TF actin backbone. Nevertheless, in addition to the C-terminal extension, another region comprised of the N-terminal and middle parts of Lmod2 presumably has a role in the Lmod2 interaction with the TF. Our data demonstrates that Lmod2 binds specifically to 3 actin subunits between the Tm junction region and Tn core (Fig 3B and 3D). This region is located in the vicinity of the TnT linker which forms a prominent density in the *activated* TF in the absence of Lmod2 (Fig 3C, insert, cyan arrow), while it is completely disordered and, hence, is not visible in the density map when Lmod2 is bound (Fig 3D, insert, cyan arrow). The disordering of the TnT linker upon interaction of Lmod2’s C-terminal extension with TF actin backbone implies that either a site within the N-terminal and/or middle parts of Lmod2 binds to TnT linker and causes its disordering, or that the binding of Lmod2’s C-terminal extension to actin causes conformational changes within the TF. The structures of TF with and without Lmod2 bound are very similar at either *relaxed* or *activated* states. Hence, it is more likely that the putative Tn-binding site in Lmod2 may interact with the TnT linker, which causes its unfolding from the helical-like conformation and spatial disordering. The possible interaction of Lmod2 with the TnT linker may explain its preferable binding to the 3 actins between the junction region and Tn core (Fig 3B and 3D).

We suggest that one of the positively charged actin-binding regions in the C-terminal extension was the WH2 domain. This is because the shape, size, and localization of the Lmod2 densities bound to the TF (Fig 3) were not consistent with that of the LRR domain, the only other potential candidate for side binding. It is structurally different from the observed densities as it has a bean-like shape and binds to a very different part of the actin molecule [41,46]. WH2 domains are well known for binding to the barbed end of F-actin [47]; however, previous studies suggest that it may also bind along the sides. It was demonstrated that expression of Lmod2[1–514] (which lacks the WH2 domain) in chick cardiomyocytes resulted in the change of Lmod2 localization to the pointed end as it was less broad and more similar to the localization of expressed Tmod1 [27]. This data can be explained by the decreased ability of Lmod2[1–514] to bind to the sides of TFs. In a separate study, only full-length but not truncated Lmod2[1–342] cosedimented with F-actin, prompting the authors to conclude that a C-terminal region, most likely the WH2 domain, plays a role in the interaction with F-actin [37]. It should be noted that in both studies, Lmod2 lacked residues in the polyP region, which shortens the linker between the newly identified binding regions from this study, thereby decreasing their interaction with F-actin while the WH2 domain retains its binding ability.

To investigate the structural features from Lmod2’s WH2 domain that may explain this result, we compared it with the WH2 domains from WASP [48], Spire [49], and Wave [48] (S7 Fig). Alignment of known structures of this domain in complex with G-actin revealed that the helical regions of the WH2 domains are very similar (S7A Fig) with RMSD values obtained for Lmod2, Spire, and WAVE’s WH2 domains as compared to that of WASP’s WH2 domain being less than 0.3 Å (S1 Table). The RMSD values for the extended chain regions were less than 1 Å for WH2 domains from Spire and Wave; however, it was ~3 Å for Lmod2’s WH2 domain. The major difference was due to the formation of a helical segment by residues IKQ (S7B Fig) in human Lmod2 which correspond to residues I537, R538, and Q539 of mouse Lmod2 used in our experiments. R or K (shown by arrows) in this region is absent in all other WH2 sequences and together with other R/K residues may form a positively charged cluster on the surface opposite to the barbed-end binding interface (S7C Fig). This cluster, when positioned correctly with other C-terminal actin-binding regions in Lmod2, could bind to negatively charged regions of F-actin. Most likely, the binding interface for the complex of Lmod2[518–543] with G-actin in the native gel-electrophoresis experiment (Fig 5D) is formed via the classical interaction between the WH2 domains and G-actin (PDB IDs 2A40, 2A3Z, 3MN5, and 5WFN) and is not the same as in a complex formed with the TF.

The 3 positively charged actin-binding sequences that were localized to the C-terminal extension of Lmod2 (Fig 5A and 5D) are in agreement with the 3 Lmod2 densities on the negatively charged patches of the actin surface revealed by cryo-EM (Fig 3B and 3D). Importantly, the distances between these sequences allow them to simultaneously bind to the three consecutive actin protomers (Fig 5E). Taking into account the likelihood of the interaction of Lmod2 with the Tn linker, we propose a model for the interaction of Lmod2 with the TF (Fig 6C). In that model, the region of Lmod2 that harbors Tm-binding site (TmBS), ABS1 and ABS2 (LRR) interacts with the TnT linker. The EDRR located between the 2 ABSs may promote the binding of Lmod2 to TFs at high Ca^2+^. Lmod2 interactions with the TnT linker directs Lmod2 C-terminal extension, which contains 3 positively charged sequences (Fig 5), to bind to the 3 adjacent actin subunits in the vicinity of the TnT linker (Fig 6C, colored ovals). Further experiments are required to experimentally evaluate this model at the atomistic level.

## Materials and methods

### Plasmid construction for Lmod2, Lmod2[1–384], and Lmod2[1–524]

For protein expression of full-length Lmod2: full-length codon-optimized mouse Lmod2 sequence flanked by an NdeI site and a His-tag on the N-terminal end as well as a SapI site at the C-terminal end was synthesized and cloned into the pUC57-Kan vector (GenScript) using KpnI and BamHI restriction enzymes. The pTXB1 *E*. *coli* expression vector was digested with NdeI and SapI. The pUC57-Kan mLmod2 construct was digested with NdeI and SapI and inserted into pTXB1 vector. The His-tag was then removed from the pTXB1 mLmod2 construct using the following primers: forward 5′-ATAACATATGTCAACATTTGGCTATCGTCGCGGTCTAAGCAAATACGAAAGTATTG-3′ and Reverse 5′-ATATGCTCTTCCGCACGCTCTGAGCGCCTCCGGC-3′. Once the sequence was confirmed using DNA sequencing, we truncated the pTXB1 mLmod2 construct (lacking the his-tag) using the following primers: mLmod2 [1–524] Forward 5′-GCGGCATATGTCAACATTTGGCTATCGTCGCGGTCTAAGCAAATACGAAAGTATTG-3′ and reverse 5′-ATATGCTCTTCCGCACGCCACGGATCTCTGAGGGGTGGACGGGC-3′ and mLmod2 [1–384] forward 5′-GCGGCATATGTCAACATTTGGCTATCGTCGCGGTCTAAGCAAATACGAAAGTATTG-3′ and reverse 5′-ATATGCTCTTCCGCAGGTGCGCAATGCTGCACCAC-3′. All sequences were confirmed using DNA sequencing.

### Protein purification

Full-length Lmod2 was purified as previously described [40]. For Lmod2[1–524] and Lmod2[1–384] expression, Rosetta 2 (DE3) pLysS competent cells (EMD Millipore) were transformed with codon-optimized mouse Lmod2, mouse Lmod2 [1–524] and [1–384] pTXB1 constructs and grown to OD 0.7 in LB media at 37°C, 250 rpm. Protein expression was induced with 1 mM IPTG for 4 h at 37°C, 250 rpm. Cells were pelleted at 5,500 × g, 4°C, for 15 min and frozen at −20°C until processing. Pellets were resuspended in B-per bacterial protein extraction reagent (Thermo Fisher Scientific) containing 1× Halt protease inhibitor cocktail (Thermo Fisher Scientific) and 5 units per ml of DNase I, and then incubated 15 min at room temperature followed by 15 min on ice. Cells were sonicated on ice for 20 s 10 times, with 20-s rests in between. The cell extract was then clarified by centrifugation at 11,500 rpm for 30 min (Sorvall RC-5B, F15S-8x50C rotor) and separated from the pellet. The supernatant was loaded into a column containing 10 ml of chitin resin (New England BioLabs) after equilibration with column buffer (50 mM Tris Buffer (pH 8.5), 500 mM NaCl, 1 mM EDTA) with 0.2% Tween 20. The columns were washed with 20 bed volumes of column buffer with Tween20 and 20 bed volumes of column buffer (minus Tween20). The proteins were cleaved from the column for 48 h using 100 mM DTT in column buffer and 1× Halt protease inhibitor cocktail (Thermo Fisher Scientific) and eluted with column buffer. The proteins were dialyzed into Lmod2 buffer (150 mM KCl, 2 mM MgCl_2_, 50 mM Tris (pH 7.5)) and ran through a second chitin column equilibrated with Lmod2 buffer to remove the remaining un-cleaved protein.

Fragments Lmod2[387–426], Lmod2[451–492], Lmod2[489–524], and Lmod2[518–543] were synthesized by the Tufts University Core Facility (Boston, Massachusetts, United States of America). Protein concentrations were determined using Pierce BCA protein assay kit (Thermo Fisher Scientific).

### Native porcine cardiac thin filaments purification

Native porcine cardiac thin filaments were prepared as described in [50]. Whole frozen porcine hearts were obtained from Pel Freez and stored at −80°C until needed. Approximately 100 to 125 grams of ventricular tissue was removed and thawed on ice. The tissue was passed through a cold meat grinder and collected in a 1 liter beaker on ice. The tissue was suspended in 400 ml of Buffer F1 (10 mM Na_2_HPO_4_, 100 mM NaCl, 5 mM MgCl_2_, 1 mM EGTA, 1 mM NaN_3_,1 mM DTT (pH 7)) containing 1% Triton-X 100 (v/v) and homogenized for 40 s using a Polytron foam-reducing homogenizer. The tissue was pelleted by spinning at 15,000 × g for 8 min. The resulting supernatant was discarded and the homogenization step was repeated once more with Buffer F1 + Triton-X and an additional 3 times using Buffer F1 without detergent. The thin filaments were extracted from the washed myofibrils by re-suspending and homogenizing twice in 180 ml of Buffer F1 + 5 mM ATP followed by spinning at 15,000 × g for 8 min. The supernatants were combined and centrifuged at 186,000 × g for 15 min. The supernatants were transferred to fresh tubes and centrifuged for 2.5 h at 186,000 × g. The pelleted crude thin filaments were gently re-suspended using a tissue homogenizer to approximately 120 ml in Dialysis Buffer (20 mM TRIS, 100 mM NaCl, 5 mM MgCl_2_, 1 mM EGTA, 1 mM NaN_3_, 1 mM DTT (pH 7.0)) and dialyzed overnight against the same. The following morning, the solution was adjusted to 20 mM TRIS, 200 mM NaCl, 2 mM ATP, 2.5 mM MgCl_2_, 1 mM EGTA, 1 mM DTT, 1 mM NaN_3_ (pH 7.0) by the addition of an equal volume of ATP Release Adjustment Buffer (20 mM TRIS, 300 mM NaCl, 1 mM EGTA, 1 mM DTT, 1 mM NaN_3_, 4 mM ATP (pH 7.0)). The solution was stirred on ice for 10 min before spinning at 186,000 × g for 15 min, discarding the pellets and spinning for another 2.5 h at the same speed. The pellets were re-suspended with Dialysis Buffer as described previously to approximately 90 ml and dialyzed overnight. The thin filaments were again diluted with an equal volume of ATP Release Adjustment Buffer, stirred on ice for 10 min and centrifuged as above. The final thin filament pellets were re-suspended to approximately 10 to 15 ml to provide a concentration of 150 to 200 mM. The concentration was determined after accounting for light scattering (OD_280_ –(OD_320_ × 1.5)) using an extinction coefficient of 0.765 mg^-1^/ml^-1^ and a molecular weight of 63,000.

### Cosedimentation of Lmod2, Lmod2[1–524], and Lmod2[1–384] with thin filaments at pCa 3.5 and pCa >8

Thin filaments were dialyzed against Buffer B (20 mM Tris-HCl (pH 7.5), 100 mM KCl, 2 mM MgCl_2_, 1 mM DTT). Full-length Lmod2 forms aggregates and loses functionality presumably due to the presence of highly disordered and oppositely charged regions. To minimize loss of functionality, reactions were kept between 0 and 4°C at all times during reaction preparation. To minimize aggregation that results in nonspecific cosedimentation, Lmod2, Lmod2[1–524], or Lmod2[1–384] were incubated with 4 mM DTT for 10 min on ice, diluted 4× for a final concentration of 1 mM DTT, then centrifuged at 213,600 × g for 30 min at 4°C prior to cosedimentation experiments. The supernatant was collected and used for cosedimentation experiments immediately. To determine the effect of Ca^2+^ on binding to thin filaments, CaCl_2_ or EGTA was added to the reaction prior to the addition of the Lmod2, Lmod2[1–524], and Lmod2[1–384]. Final CaCl_2_ concentration was 0.3 mM for pCa 3.5 and final EGTA concentration was 2 mM for pCa >8. pCa was calculated using an online tool called CHELATOR [51]. The calculation was done using buffer components and ionic strength. Ionic strength was calculated using an online Ionic Strength Calculator (http://calistry.org/calculate/ionic-strength-calculator). Lmod2, Lmod2[1–524], or Lmod2[1–384] at pCa 3.5 and pCa >8 was added to thin filaments at increasing concentrations. Immediately after all components were mixed, the mixtures were centrifuged at 213,600 × g for 30 min at 4°C. Pellets were washed with Buffer B (20 mM Tris-HCl (pH 7.5), 100 mM KCl, 2 mM MgCl_2_, 1 mM DTT) and reconstituted in 8M urea for analysis by SDS-PAGE. Pellets were run on 10% polyacrylamide gels, then stained with Coomassie Brilliant Blue R-250. Images were captured using Molecular Imager ChemiDoc XRS^+^ (Bio-Rad, Hercules, California, USA). Quantitative analysis of the band densities was done using Bio-Rad’s ImageLab software.

### Pyrene-actin polymerization assays

Pyrene-actin fluorescence was measured using an Agilent Cary Eclipse fluorescence spectrometer (Santa Clara, California, USA). Rabbit skeletal actin (Cytoskeleton, Inc. Cat. # AKL99-A) and pyrene-labeled rabbit skeletal actin (Cytoskeleton, Inc. Cat. # AP05-A) were reconstituted and further diluted according to the manufacturer’s instructions; 1.5 μM G-actin was used (10% pyrenyl-actin, 90% unlabeled actin) to measure nucleation of actin polymerization in the absence (control) or presence of different concentrations of wild-type Lmod2, Lmod2[1–524], and Lmod2[1–384]. Actin was incubated with Lmod2 (wild-type or mutant) for 5 min at room temperature previous to the addition of 1× polymerization buffer (25 mM Imidazole (pH 7.0), 100 mM KCl, 2 mM MgCl_2_, 1 mM EGTA, 2 mM Tris–HCl, 0.2 mM CaCl_2_, 0.2 mM ATP, and 0.5 mM DTT). Exponential growth curves to maximum were fitted to the polymerization data using SigmaPlot 12.0. Initial rates were calculated as the first derivatives at time zero and normalized to control (to account for spontaneous actin polymerization) [52,53].

### Secondary structure predictions

Secondary structure prediction of the C-terminal extension of Lmod2 was done using Jpred4: A Protein Secondary Structure Prediction Server (https://www.compbio.dundee.ac.uk/jpred/). An additional secondary structure prediction was done using AlphaFold [54]. Isoelectric points (pI) of synthetic fragments were predicted using [55]. The predicted pIs were 12.02 for Lmod2[387–426], 10.86 for Lmod2[451–492], 10.16 for Lmod2[489–524], and 11.82 for Lmod2[518–543].

### Circular dichroism (CD)

CD spectra were recorded using an Aviv model 420 spectropolarimeter (Lakewood, New Jersey) in a 1 mm cuvette at 20°C. The measurements were done in 10 mM sodium phosphate (pH 7.0) containing 100 mM NaCl. Concentrations of Lmod2[387–426], Lmod2[451–492], and Lmod2[489–524] were determined to be 25 μM, 19 μM, and 23 μM, respectively.

### Native gel electrophoresis

To test binding of the synthetic fragments to G-actin, we used native polyacrylamide gel electrophoresis in 9% polyacrylamide gels polymerized in the presence of 10% glycerol without SDS as in [56]. Each of the 3 C-terminal Lmod2 fragments (Lmod2[387–426], Lmod2[451–492], and Lmod2[489–524]) were mixed with 5 μM G-actin at 1:2, 1:1, 1.5:1, and 2:1 molar ratios. Lmod2[518–543], containing the WH2 domain, was used as a positive control at a ratio of 2:1 [41]. Isoelectric points were calculated using the ProtParam tool at Expasy (https://web.expasy.org/protparam/).

### Molecular dynamics simulations (MDS)

Polypeptide structure editing and visualization was done in UCSF Chimera [57]. The initial structure of the C-terminal extension of Lmod2 was created using backbone dihedral angles predicted using AlphaFold [54]. The structure was neutralized with Cl^-^ ions and placed in a box of OPC water molecules [58]. The MDS was performed using AMBER 22 [59] with the ff19SB force field and periodic boundary conditions. The structure was minimized and relaxed to avoid atomic clashes. After minimization, the structure was slowly heated from 100 K to 300 K while introducing pressure control with Monte Carlo barostat. For our production run, there was no pressure control. The temperature was controlled by a Langevin thermostat using a 3-ps^-1^ collision frequency. Hydrogens were constrained using SHAKE. MDS production run was 200 ns.

To determine the frames from MDS to use in creating an average structure, we plotted the RMSD for the length of the production run and determined the time range in which the structure was most stable (40 ns to 82 ns). End-to-end distances were determined using α-carbons of residues of interest.

### Statistical analysis

Statistical analysis was conducted for cosedimentation of full-length Lmod2 and thin filaments at pCa >8 and pCa 3.5 using one-way ANOVA in SigmaPlot12.

### Cryo-EM

A total of 1.5 μl of 1 μM native TFs in B-buffer with Ca^2+^ (0.3 mM CaCl_2_) or without Ca^2+^ (2 mM EGTA) conditions was applied onto the glow discharged lacey carbon grid and gently blotted by hand and immediately mixed with 2 μl of 4 μM Lmod2. After 1-min incubation, the grid was blotted with Whatman Grade 1 Filter Paper for 4 s and vitrified in a Vitrobot Mark IV (FEI, Inc.). Summary for imaging conditions and image reconstruction is provided in S2 Table. Image analysis was performed using RELION [60], while modeling was done using UCSF Chimera [61]. Experimental details are provided in the Supporting Information Materials and Methods.

## Supporting information

S1 Fig Cosedimentation of cardiac TFs with Lmod2, Lmod2[1–524], and Lmod2[1–384] at high Ca2+. (A) Full-length Lmod2 sequence (residues 1–550) with positions of the truncations shown. (B) 1.5 μM TF was pelleted (213,600 × g) with a range of Lmod2 concentrations (0.25–2.0 μM) at pCa 3.5. Lmod to actin density ratios in pellets were measured for Lmod2[1–384], Lmod2[1–524], and full-length Lmod2 by subtracting the density of the Lmod band that pelleted in the absence of TF at each concentration and dividing by the density of the actin band. (C) Representative SDS-PAGE is shown for Lmod2[1–384]. Concentration of Lmod2[1–384] is shown below the gel. The data underlying this figure can be found in S1 Data.
(TIF)

S2 Fig 3D reconstruction of the native cTF in Ca2+-free conditions in the presence of Lmod2. (A, B) Overlapping cTF segments were automatically selected and extracted from the cryo-EM micrographs to use in 2D classification (B). (C) The best segments from the 2D classification were sorted to select segments possessing the Tn tandem near the center of the filament (red box). (D) Segments selected in (C) were further classified into 3 classes based on occupancy of the Tn tandem and the ones that were intact (red box) were used for 3D refinement (E). (F) The global resolution determined by the FSC was determined to be ~6.2 Å. The data underlying this figure can be found in S3 Data. (G) The local resolution map shows that the best resolution of 5.05 Å is within the TF backbone, while the resolution of the actin surface, Lmod2 density and Tm density are resolved to 9 Å or better. The lowest resolution was 12.37 Å at the terminal actins.
(TIF)

S3 Fig 3D reconstruction of the the native cTF in Ca2+-saturated conditions in the presence of Lmod2. (A, B) Overlapping cTF segments were manually selected and extracted from the cryo-EM micrographs to use in 2D classification (B). (C) The best segments from the 2D sorting were classified based on the position of Tn to select segments possessing the Tn pair proximal to the center of the filament (red box). (D) Segments selected in (C) were sorted by integrity of the Tn complex to select particles with intact Tn pair (red box) for the following 3D refinement (E). (F) The global resolution of ~6.1 Å was determined by Fourier Shell Correlation (FSC) 0.143 criterion. The data underlying this figure can be found in S4 Data. (G) The local resolution map shows that the best resolution of 5.5 Å is within the TF backbone, while the resolution of the actin surface, Lmod2 density and Tm density are resolved to 9 Å or better. The lowest resolution was 13.95 Å at the distal actins.
(TIF)

S4 Fig Actin nucleation is higher in the presence of Lmod2 [1–524] when compared to Lmod2[1–384]. (A) Representative curves of pyrene actin fluorescence (A.U: arbitrary units) over time (min) in the presence of 100 nM Lmod2, Lmod2[1–524], and Lmod2[1–384]. Control is 1.5 μM G-actin alone. The data underlying this figure can be found in S2 Data. (B) Concentration-dependent actin polymerization rates in the presence of Lmod2 (red), Lmod2[1–524] (blue), or Lmod2[1–384] (green). Actin polymerization rates relative to the control (R_exp_/R_control_) were calculated as the first derivatives at time zero after exponential fit. All values are shown as mean ± SD; *P* < 0.05 was considered significant. Statistically significant values when compared to mLmod2 (*). Statistically significant values when comparing mLmod2[1–524] and mLmod2[1–384] (^&^). *P* < 0.05 (*), *P* < 0.01 (**), *P* < 0.001 (***), *P* < 0.0001 (****). *P* < 0.05 (^&^), *P* < 0.01 (^&&^), *P* < 0.001 (^&&&^), *P* < 0.0001 (^&&&&^), ns: not significant. One-way ANOVA followed by Sidak’s multiple comparisons test. *n* = 3. The data underlying this figure can be found in S2 Data.
(TIF)

S5 Fig MDS results for the C-terminal extension. (A) End-to-end distance for the entire C-terminal extension (res. 373–550). (B) End-to-end distance for the polyP region (res. 427–450). (C) End-to-end distance for residues 489–524. The data underlying this figure can be found in S2 Data.
(TIF)

S6 Fig Lmod2 binding to TF actin backbone blocks myosin binding sites on the TF. (A) Myosin heads (yellow ribbons and gray transparent surfaces from PDB 7JH7 [4]) clashes (red arrows) with Lmod2 (purple surfaces) on the surface of the actin filament (tan ribbons). Tm is shown as orange ribbons, while TnT1 is depicted as cyan ribbons. (B–D) When Lmod2 density on the surface of TF in the relaxed (B) and activated (C) states (purple meshwork) is protruded on the model of the TF (D), only actin protomers at or above the Tm junction region (black bracket) are affected (red circles), while the lower 2 sites are available for actomyosin interactions (green circles). Actin subunits are shown as tan ribbons, Tn complex is shown as green ribbons, Tm is shown as yellow ribbons, while TnT1 is shown as cyan ribbons.
(TIF)

S7 Fig Sequence and structure alignment of WH2 domains. **(A)** Structure alignments of 4 WH2 domains, WASP (green), Spire (blue), WAVE (pink), and hLmod2 (tan), in complex with actin monomers (in several shades of gray). The top panel shows aligned WH2 domain locations on aligned actin monomers. The bottom panel shows the actin monomers rotated 50° about the x-axis and magnified to better show the WH2 domain alignments. The numbers indicate actin subdomains. **(B)** Sequence alignment of the 4 WH2 domains used for structure alignments and the corresponding WH2 sequence of mLmod2. Bold/underline are helical regions from the structures, underline only is a helical region from secondary structure prediction, highlighted in yellow are residues in WASP conserved in more than 50% of the WH2 sequences [5], residues in red are those in WASP conserved in more than 80% of WH2 sequences, and highlighted in cyan are positively charged residues in the WH2 sequence of Lmod2. **(C)** Three views of surface maps of aligned WH2 domains from hLmod2 and WASP depicting positive (blue) and negative (red) charges. The middle panel is rotated 50° about the x-axis from the top panel and then the bottom panel is rotated another 20° from the middle panel to better see important charge differences between hLmod2 and WASP WH2 domains.
(TIF)

S1 Raw images Images of SDS-PAGE gels. Raw images of gels with marked lanes are provided for Figs 2A (page 1), 2B (page 2), 5D panel 1, 2, and 3 (pages 3, 4, and 5, respectively), S1C (page 6), and S1B (pages 7 and 8).
(PDF)

S1 Data Excel Tables with raw data. Raw numerical data used to plot data to calculate statistics are provided for the following Figs: 2C, 4A, 4B, 5B, S1B, S4A, S4B, S5A, S1B, and S1C as excel sheets. Each sheet contains appropriate columns names.
(XLSX)

S2 Data Contains raw numbers in XML format calculated by RELION [1] for the Fourier Shell Correlation plot shown in S2F Fig. (XML)

S3 Data Contains raw numbers in XML format calculated by RELION [1] for the Fourier Shell Correlation plot shown in S3F Fig. (XML)

S1 Table RMSD values of the backbone atoms of aligned WH2 domains in complex with actin monomers as compared to the α-helical (residues 1–10) and disordered (residues 11–17) regions of WASP WH2 domain (from same PDBs as given in S7B Fig). (DOCX)

S2 Table Data collection and refinement statistics. (DOCX)

## Abbreviations

ABS1: actin-binding site 1
ABS2: actin-binding site 2
CD: circular dichroism
DCM: dilated cardiomyopathy
MDS: molecular dynamics simulation
MyBP-C: myosin-binding protein C
LRR: leucine-rich repeat
TmBS: Tm-binding site
WH2: Wiskott–Aldrich homology 2
