# Supplementary figures and images for: Interaction of cardiac leiomodin with the native cardiac thin filament
Source: PLoS Biol. 2025 Jan 30;23(1):e3003027. doi: 10.1371/journal.pbio.3003027 (PMC11813103; doi:10.1371/journal.pbio.3003027)

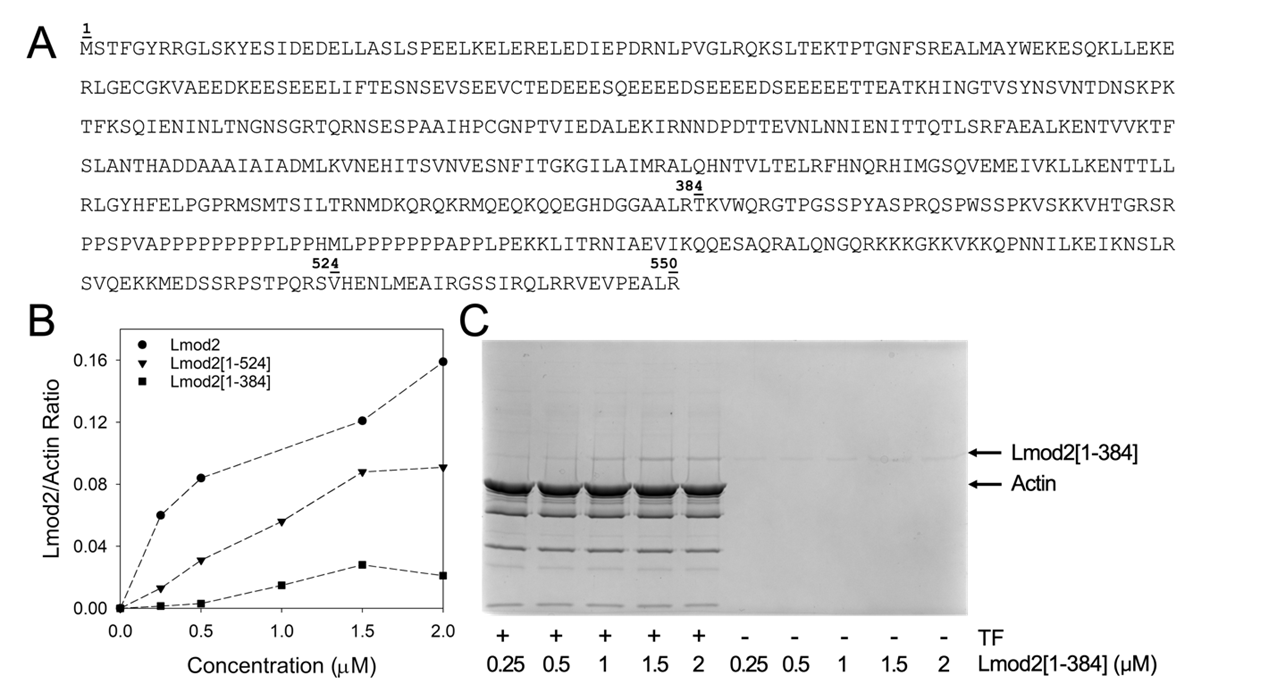

Supplement: S1 Fig — (A) Full-length Lmod2 sequence (residues 1–550) with positions of the truncations shown. (B) 1.5 μM TF was pelleted (213,600 × g) with a range of Lmod2 concentrations (0.25–2.0 μM) at pCa 3.5. Lmod to actin density ratios in pellets were measured for Lmod2[1–384], Lmod2[1–524], and full-length Lmod2 by subtracting the density of the Lmod band that pelleted in the absence of TF at each concentration and dividing by the density of the actin band. (C) Representative SDS-PAGE is shown for Lmod2[1–384]. Concentration of Lmod2[1–384] is shown below the gel. The data underlying this figure can be found in S1 Data. (TIF) [file pbio.3003027.s001.tif]

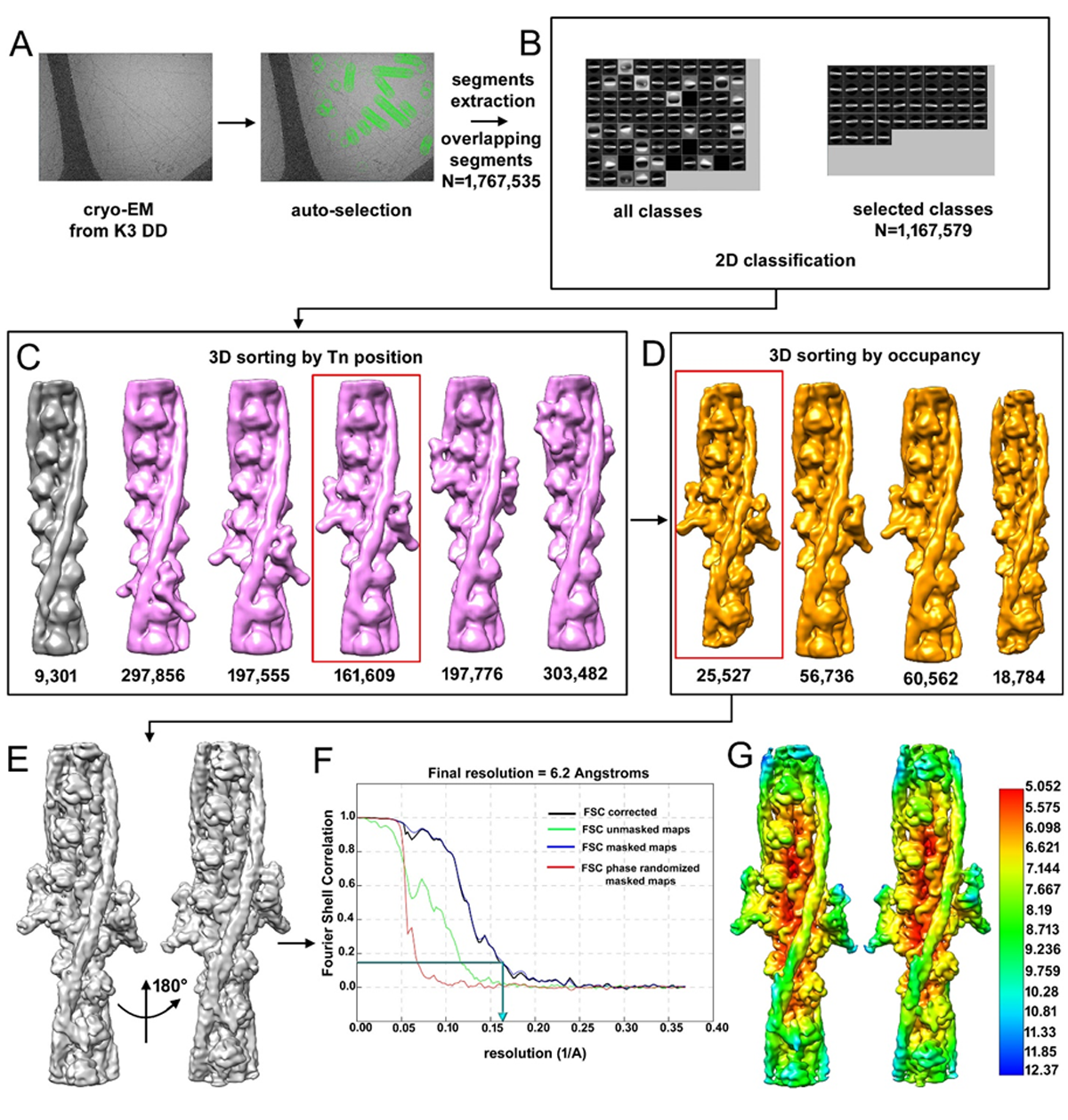

Supplement: S2 Fig — (A, B) Overlapping cTF segments were automatically selected and extracted from the cryo-EM micrographs to use in 2D classification (B). (C) The best segments from the 2D classification were sorted to select segments possessing the Tn tandem near the center of the filament (red box). (D) Segments selected in (C) were further classified into 3 classes based on occupancy of the Tn tandem and the ones that were intact (red box) were used for 3D refinement (E). (F) The global resolution determined by the FSC was determined to be ~6.2 Å. The data underlying this figure can be found in S3 Data. (G) The local resolution map shows that the best resolution of 5.05 Å is within the TF backbone, while the resolution of the actin surface, Lmod2 density and Tm density are resolved to 9 Å or better. The lowest resolution was 12.37 Å at the terminal actins. (TIF) [file pbio.3003027.s002.tif]

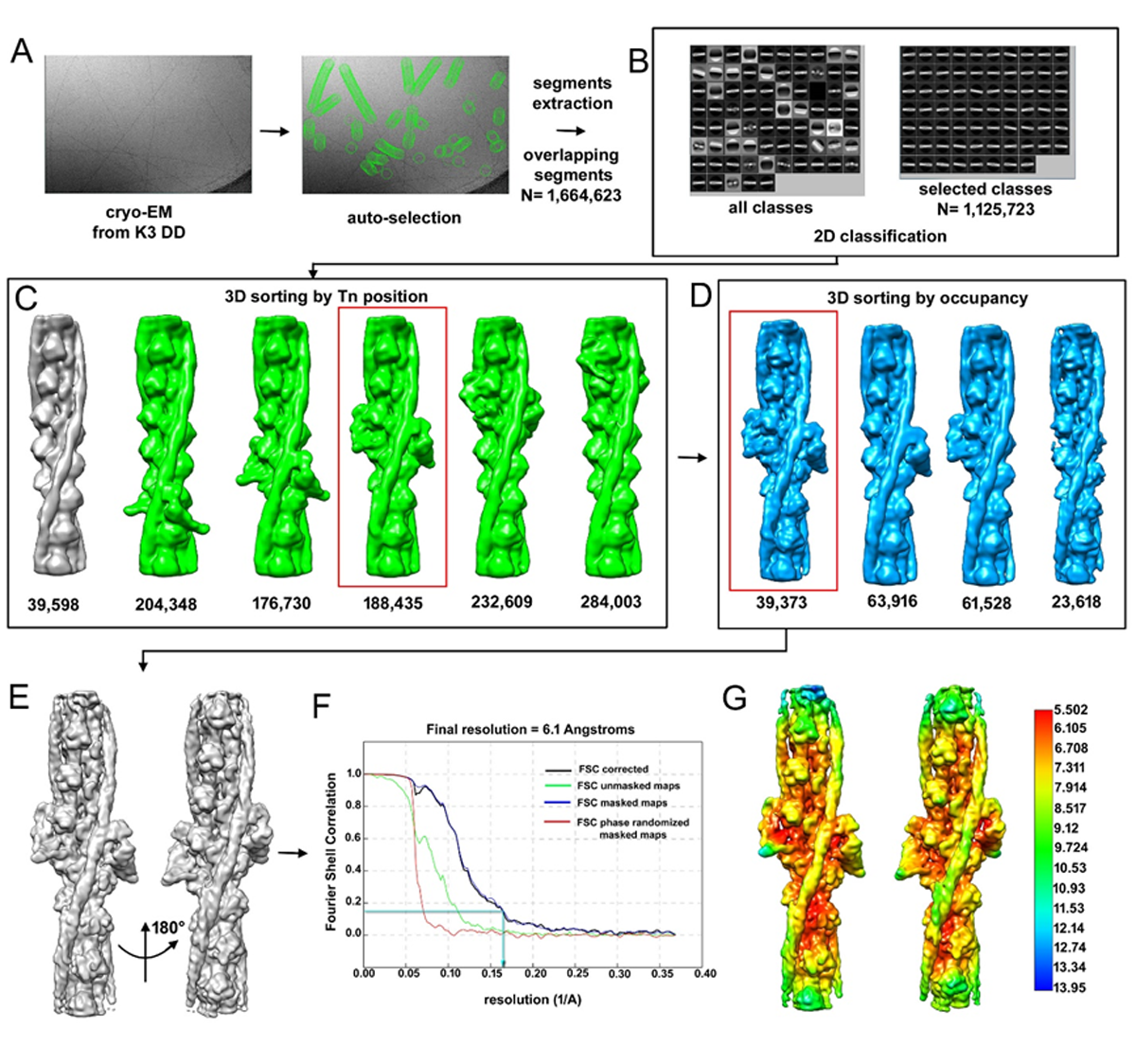

Supplement: S3 Fig — (A, B) Overlapping cTF segments were manually selected and extracted from the cryo-EM micrographs to use in 2D classification (B). (C) The best segments from the 2D sorting were classified based on the position of Tn to select segments possessing the Tn pair proximal to the center of the filament (red box). (D) Segments selected in (C) were sorted by integrity of the Tn complex to select particles with intact Tn pair (red box) for the following 3D refinement (E). (F) The global resolution of ~6.1 Å was determined by Fourier Shell Correlation (FSC) 0.143 criterion. The data underlying this figure can be found in S4 Data. (G) The local resolution map shows that the best resolution of 5.5 Å is within the TF backbone, while the resolution of the actin surface, Lmod2 density and Tm density are resolved to 9 Å or better. The lowest resolution was 13.95 Å at the distal actins. (TIF) [file pbio.3003027.s003.tif]

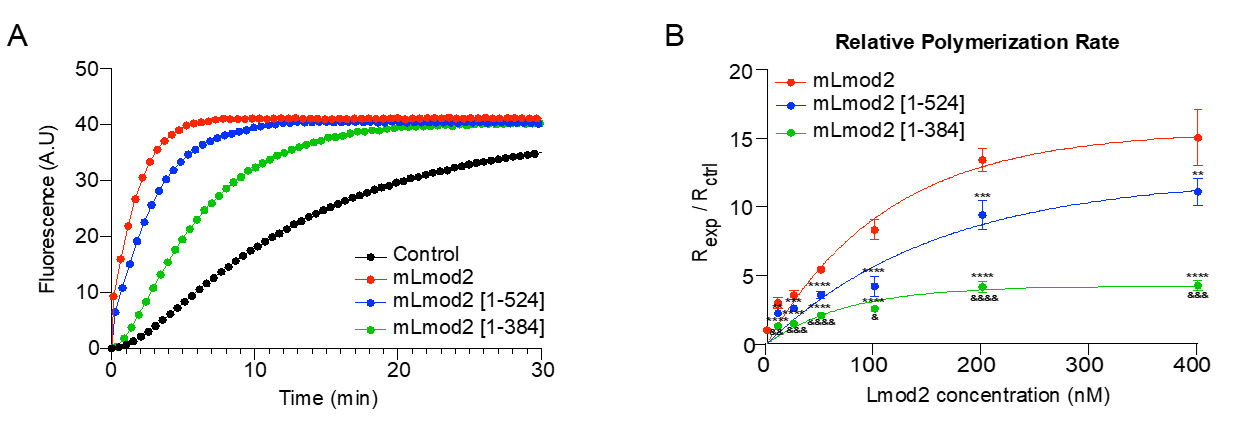

Supplement: S4 Fig — (A) Representative curves of pyrene actin fluorescence (A.U: arbitrary units) over time (min) in the presence of 100 nM Lmod2, Lmod2[1–524], and Lmod2[1–384]. Control is 1.5 μM G-actin alone. The data underlying this figure can be found in S2 Data. (B) Concentration-dependent actin polymerization rates in the presence of Lmod2 (red), Lmod2[1–524] (blue), or Lmod2[1–384] (green). Actin polymerization rates relative to the control (Rexp/Rcontrol) were calculated as the first derivatives at time zero after exponential fit. All values are shown as mean ± SD; P < 0.05 was considered significant. Statistically significant values when compared to mLmod2 (*). Statistically significant values when comparing mLmod2[1–524] and mLmod2[1–384] (&). P < 0.05 (*), P < 0.01 (**), P < 0.001 (***), P < 0.0001 (****). P < 0.05 (&), P < 0.01 (&&), P < 0.001 (&&&), P < 0.0001 (&&&&), ns: not significant. One-way ANOVA followed by Sidak’s multiple comparisons test. n = 3. The data underlying this figure can be found in S2 Data. (TIF) [file pbio.3003027.s004.tif]

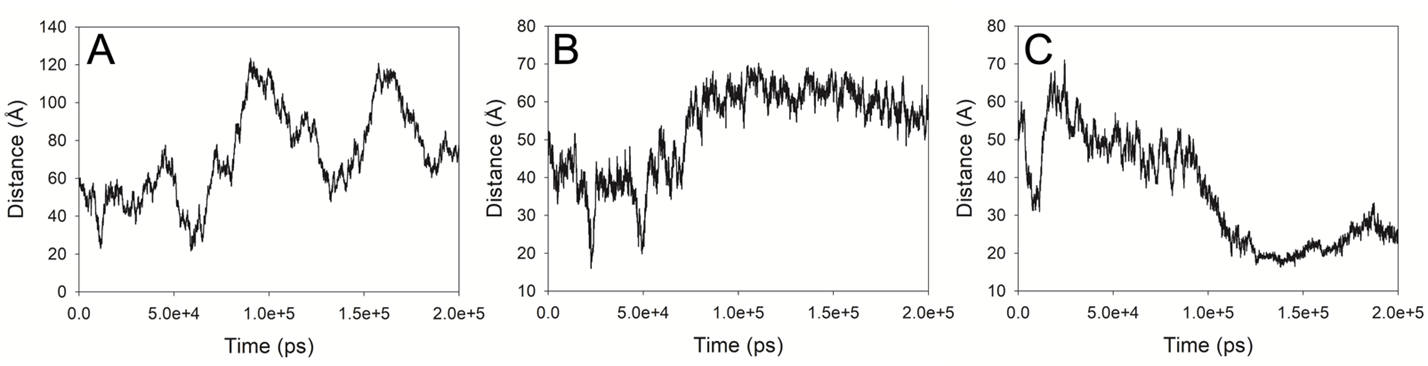

Supplement: S5 Fig — (A) End-to-end distance for the entire C-terminal extension (res. 373–550). (B) End-to-end distance for the polyP region (res. 427–450). (C) End-to-end distance for residues 489–524. The data underlying this figure can be found in S2 Data. (TIF) [file pbio.3003027.s005.tif]

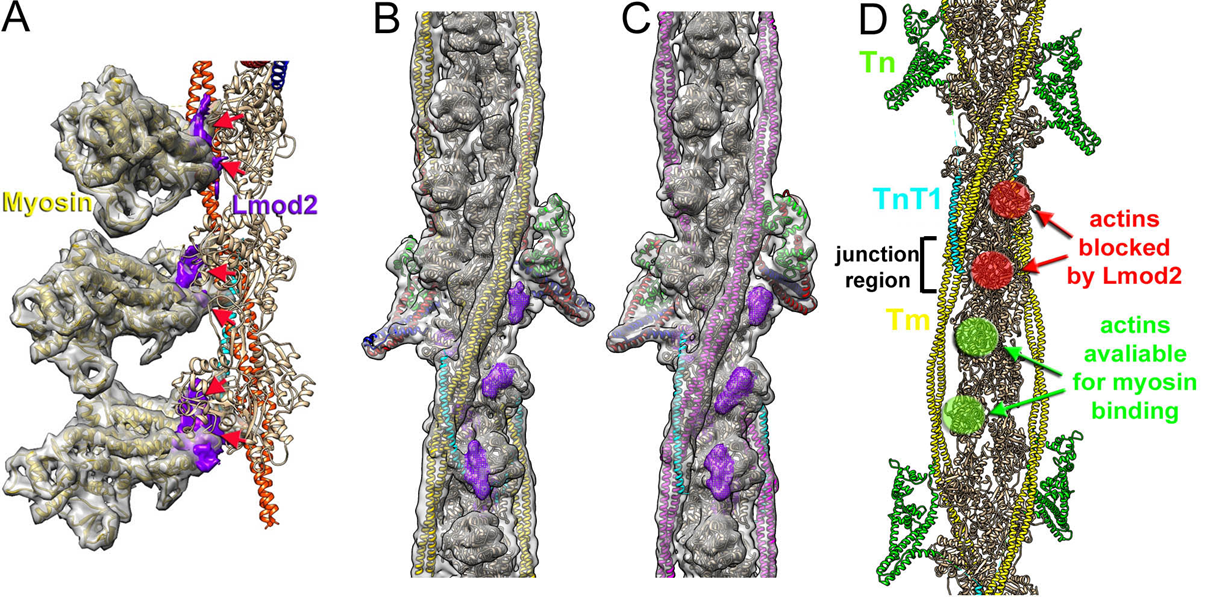

Supplement: S6 Fig — (A) Myosin heads (yellow ribbons and gray transparent surfaces from PDB 7JH7 [4]) clashes (red arrows) with Lmod2 (purple surfaces) on the surface of the actin filament (tan ribbons). Tm is shown as orange ribbons, while TnT1 is depicted as cyan ribbons. (B–D) When Lmod2 density on the surface of TF in the relaxed (B) and activated (C) states (purple meshwork) is protruded on the model of the TF (D), only actin protomers at or above the Tm junction region (black bracket) are affected (red circles), while the lower 2 sites are available for actomyosin interactions (green circles). Actin subunits are shown as tan ribbons, Tn complex is shown as green ribbons, Tm is shown as yellow ribbons, while TnT1 is shown as cyan ribbons. (TIF) [file pbio.3003027.s006.tif]

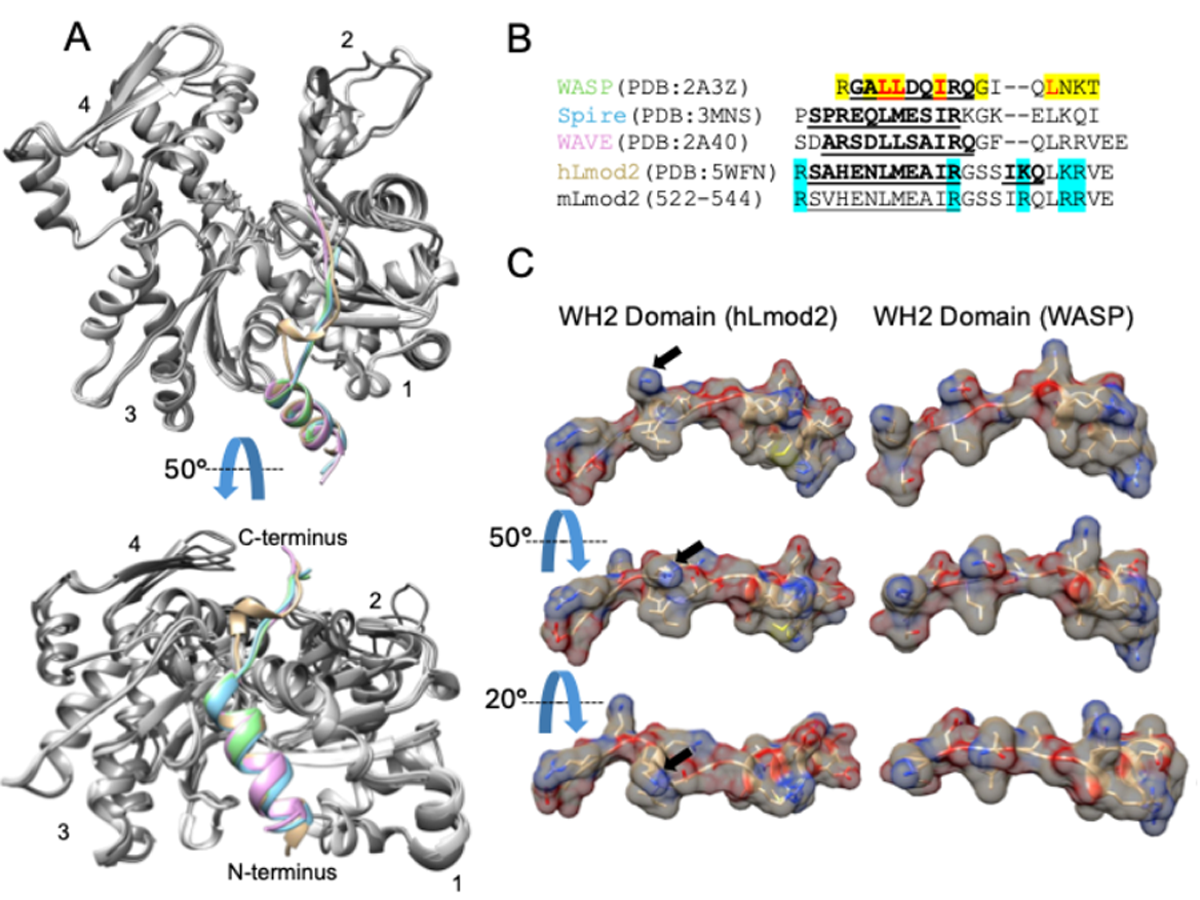

Supplement: S7 Fig — (A) Structure alignments of 4 WH2 domains, WASP (green), Spire (blue), WAVE (pink), and hLmod2 (tan), in complex with actin monomers (in several shades of gray). The top panel shows aligned WH2 domain locations on aligned actin monomers. The bottom panel shows the actin monomers rotated 50° about the x-axis and magnified to better show the WH2 domain alignments. The numbers indicate actin subdomains. (B) Sequence alignment of the 4 WH2 domains used for structure alignments and the corresponding WH2 sequence of mLmod2. Bold/underline are helical regions from the structures, underline only is a helical region from secondary structure prediction, highlighted in yellow are residues in WASP conserved in more than 50% of the WH2 sequences [5], residues in red are those in WASP conserved in more than 80% of WH2 sequences, and highlighted in cyan are positively charged residues in the WH2 sequence of Lmod2. (C) Three views of surface maps of aligned WH2 domains from hLmod2 and WASP depicting positive (blue) and negative (red) charges. The middle panel is rotated 50° about the x-axis from the top panel and then the bottom panel is rotated another 20° from the middle panel to better see important charge differences between hLmod2 and WASP WH2 domains. (TIF) [file pbio.3003027.s007.tif]
