# Supplementary material for: Interaction of cardiac leiomodin with the native cardiac thin filament
Source: PLoS Biol. 2025 Jan 30;23(1):e3003027. doi: 10.1371/journal.pbio.3003027 (PMC11813103; doi:10.1371/journal.pbio.3003027)
Supplement: S1 Raw images — Raw images of gels with marked lanes are provided for Figs 2A (page 1), 2B (page 2), 5D panel 1, 2, and 3 (pages 3, 4, and 5, respectively), S1C (page 6), and S1B (pages 7 and 8). (PDF) [file pbio.3003027.s008.pdf]

Fig. 2A

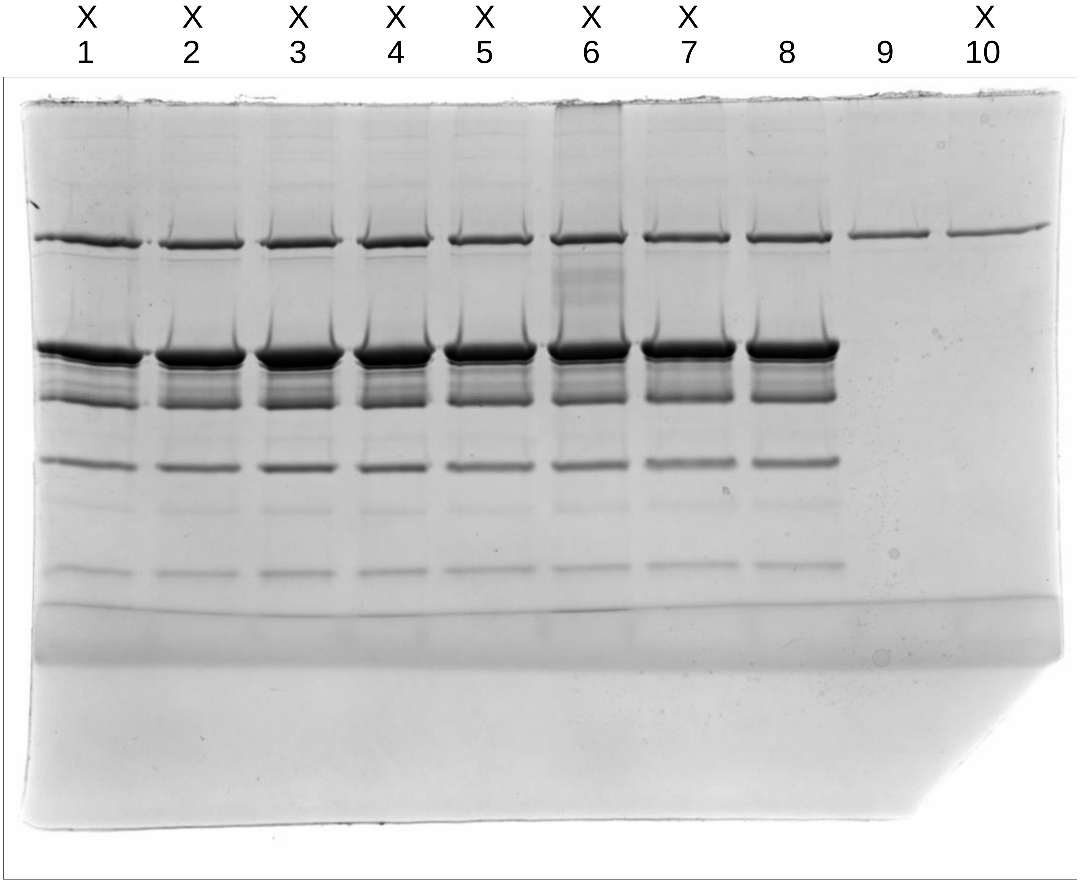

1-8: 1.5 μM Lmod2, 1 μM thin filament, pCa>8
9,10: 1.5 μM Lmod2, pCa>8

Image captured using Molecular Imager ChemiDoc XRS+ (Bio-Rad)

Fig. 2B

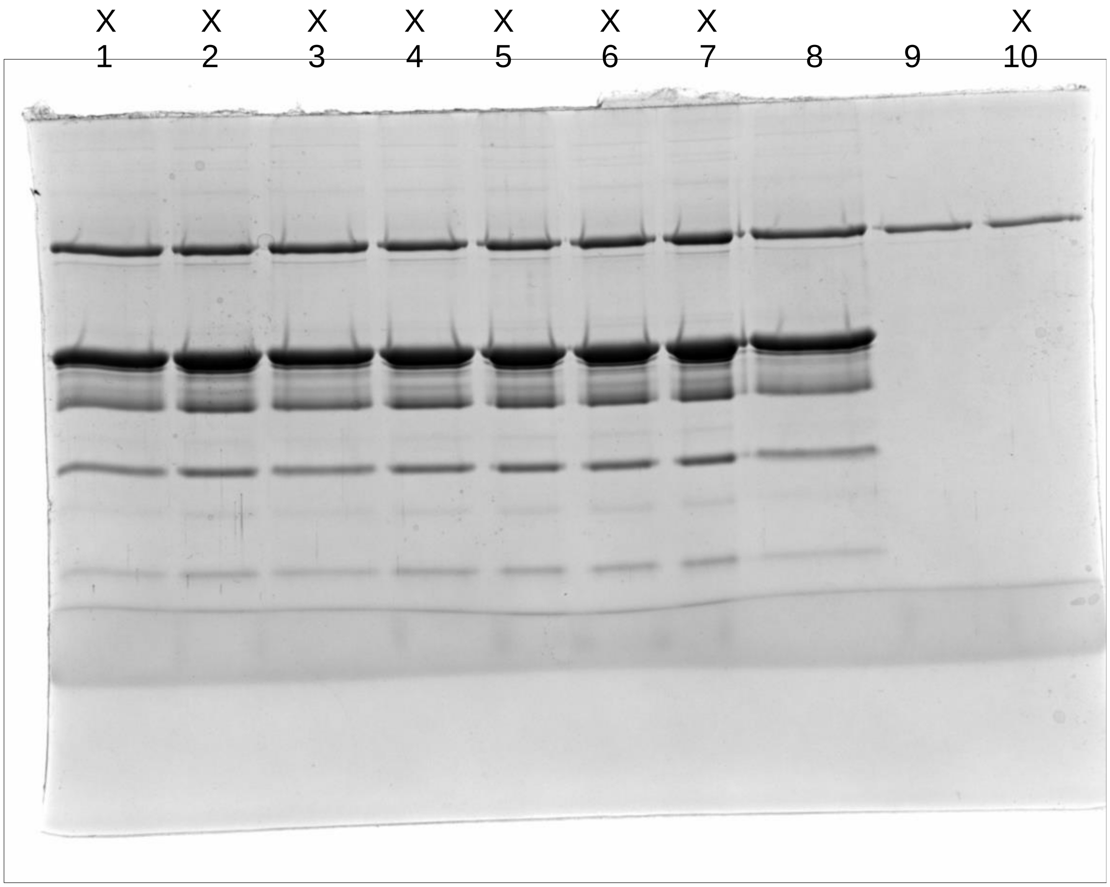

1-8: 1.5 μM Lmod2, 1 μM thin filament, pCa 3.5
9,10: 1.5 μM Lmod2, pCa 3.5

Image captured using Molecular Imager ChemiDoc XRS+ (Bio-Rad)

Fig. 5D panels 1 and 4

Panel 1: Lanes 3-8
Panel 4: Lanes 1-3

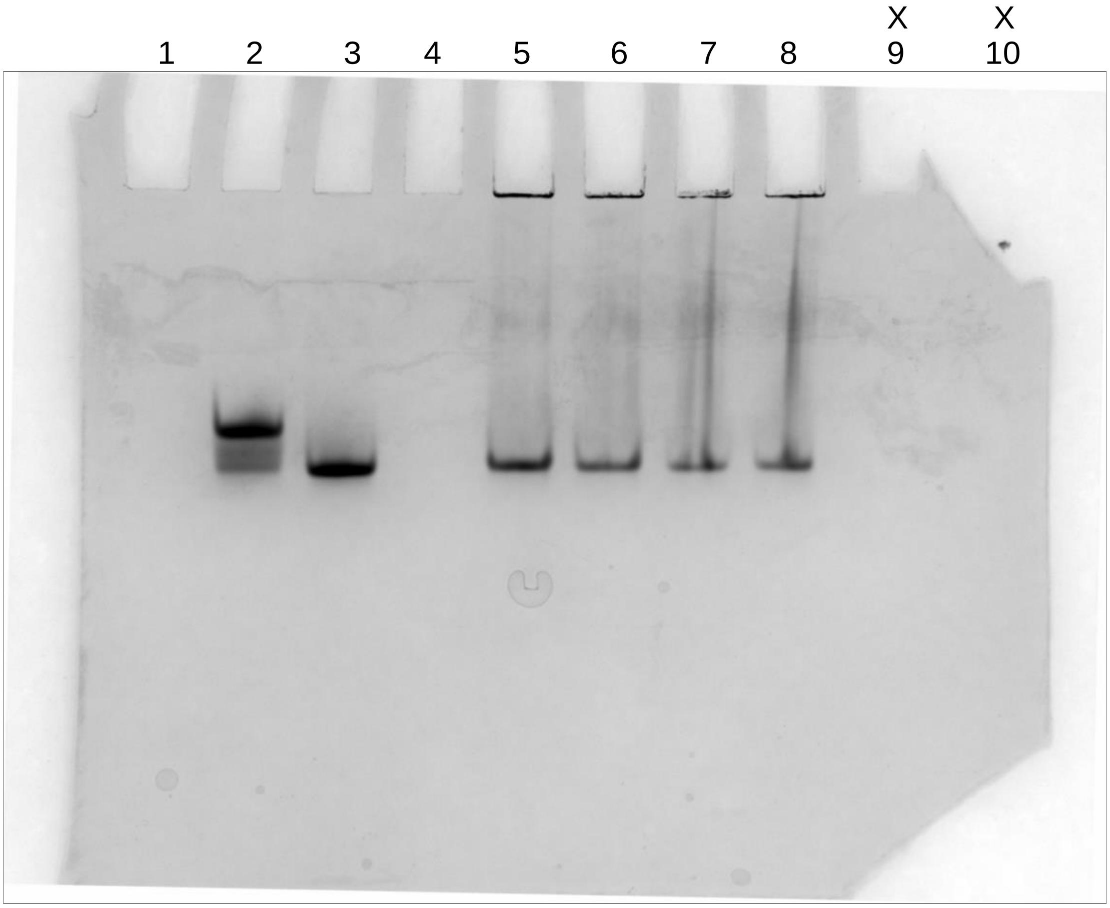

1. 10 μM Lmod2[518-543]
2. 10 μM Lmod2[518-543], 5 μM G-actin
3. 5 μM G-actin
4. 10 μM Lmod2[387-426]
5. 2.5 μM Lmod2[387-426], 5 μM G-actin
6. 5 μM Lmod2[387-426], 5 μM G-actin
7. 7.5 μM Lmod2[387-426], 5 μM G-actin
8. 10 μM Lmod2[387-426], 5 μM G-actin
9. Empty
10. Empty

Image captured using Molecular Imager ChemiDoc XRS+ (Bio-Rad)

Fig. 5D panel 2

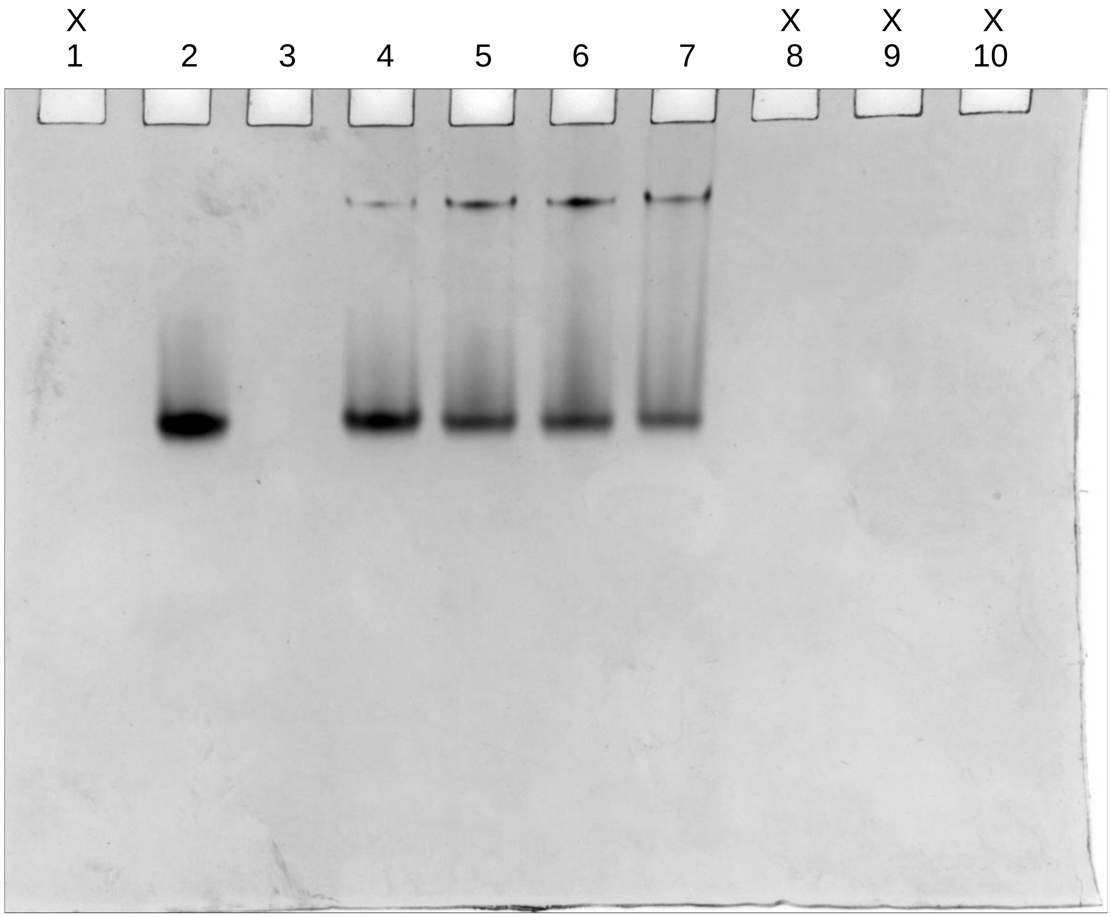

1. Empty
2. 5 μM G-actin
3. 10 μM Lmod2[451-492]
4. 2.5 μM Lmod2[451-492], 5 μM G-actin
5. 5 μM Lmod2[451-492], 5 μM G-actin
6. 7.5 μM Lmod2[451-492], 5 μM G-actin
7. 10 μM Lmod2[451-492], 5 μM G-actin
8. Empty
9. Empty
10. Empty

Image captured using Molecular Imager ChemiDoc XRS^{+} (Bio-Rad)

Fig. 5D panel 3

X X X X

1 2 3 4 5 6 7 8 9 10

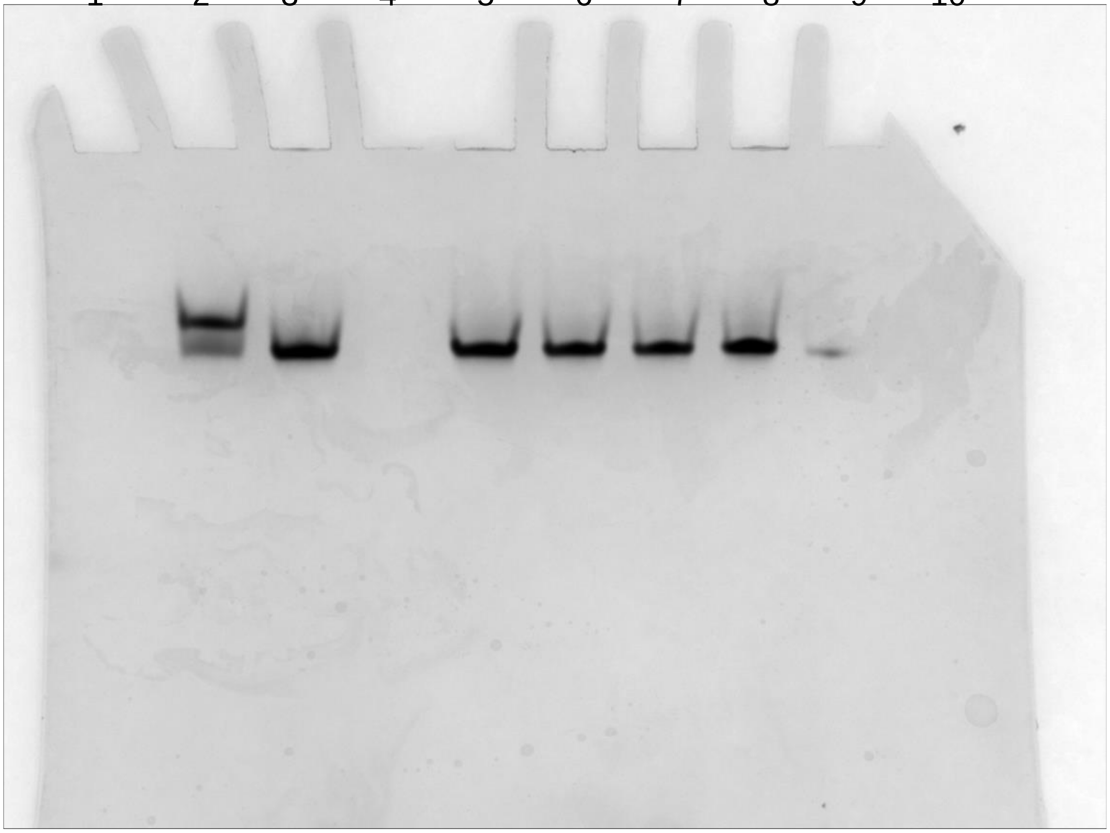

1. 10 μM Lmod2[518-543]
2. 10 μM Lmod2[518-543], 5 μM G-actin
3. 5 μM G-actin
4. 10 μM Lmod2[489-524]
5. 2.5 μM Lmod2[489-524], 5 μM G-actin
6. 5 μM Lmod2[489-524], 5 μM G-actin
7. 7.5 μM Lmod2[489-524], 5 μM G-actin
8. 10 μM Lmod2[489-524], 5 μM G-actin
9. Empty
10. Empty

Image captured using Molecular Imager ChemiDoc XRS^{+} (Bio-Rad)

Fig. S1C

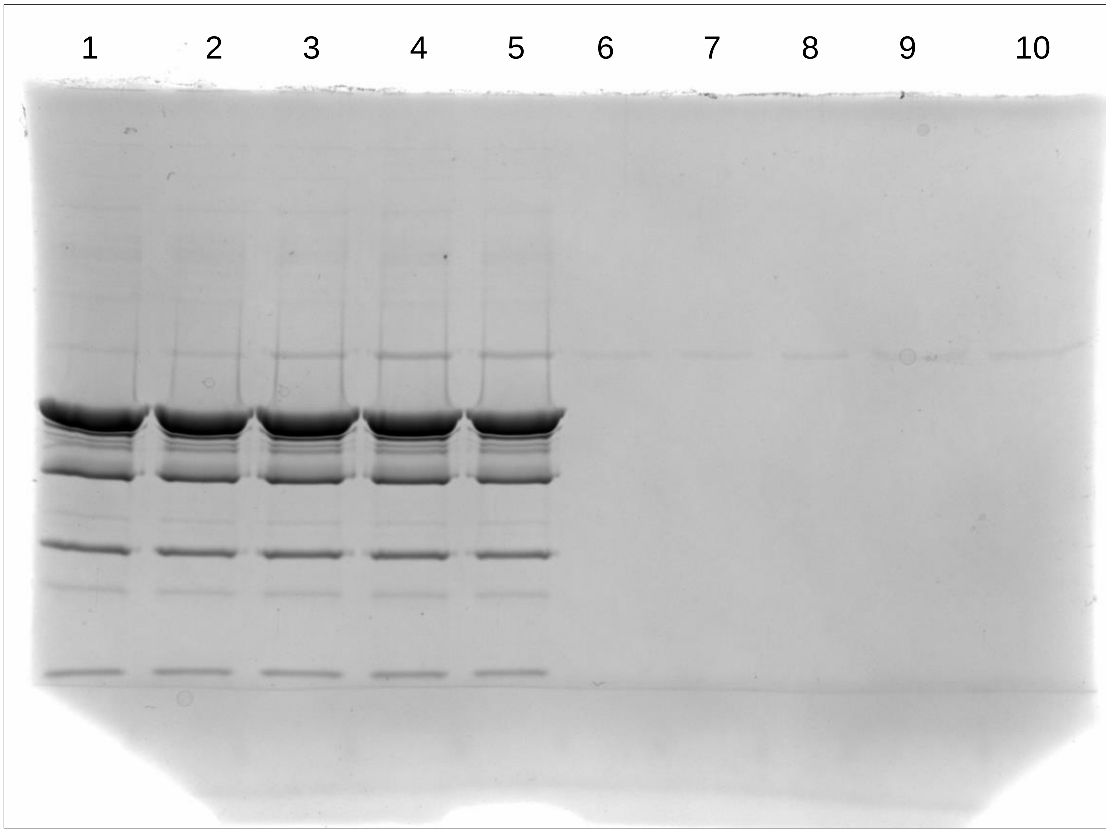

1. 1.5 μM thin filament, 0.25 μM Lmod2[1-384]
2. 1.5 μM thin filament, 0.5 μM Lmod2[1-384]
3. 1.5 μM thin filament, 1 μM Lmod2[1-384]
4. 1.5 μM thin filament, 1.5 μM Lmod2[1-384]
5. 1.5 μM thin filament, 2 μM Lmod2[1-384]
6. 0.25 μM Lmod2[1-384]
7. 0.5 μM Lmod2[1-384]
8. 1 μM Lmod2[1-384]
9. 1.5 μM Lmod2[1-384]
10. 2 μM Lmod2[1-384]

Image captured using Molecular Imager ChemiDoc XRS^{+} (Bio-Rad)

Data used to make Figure S1B

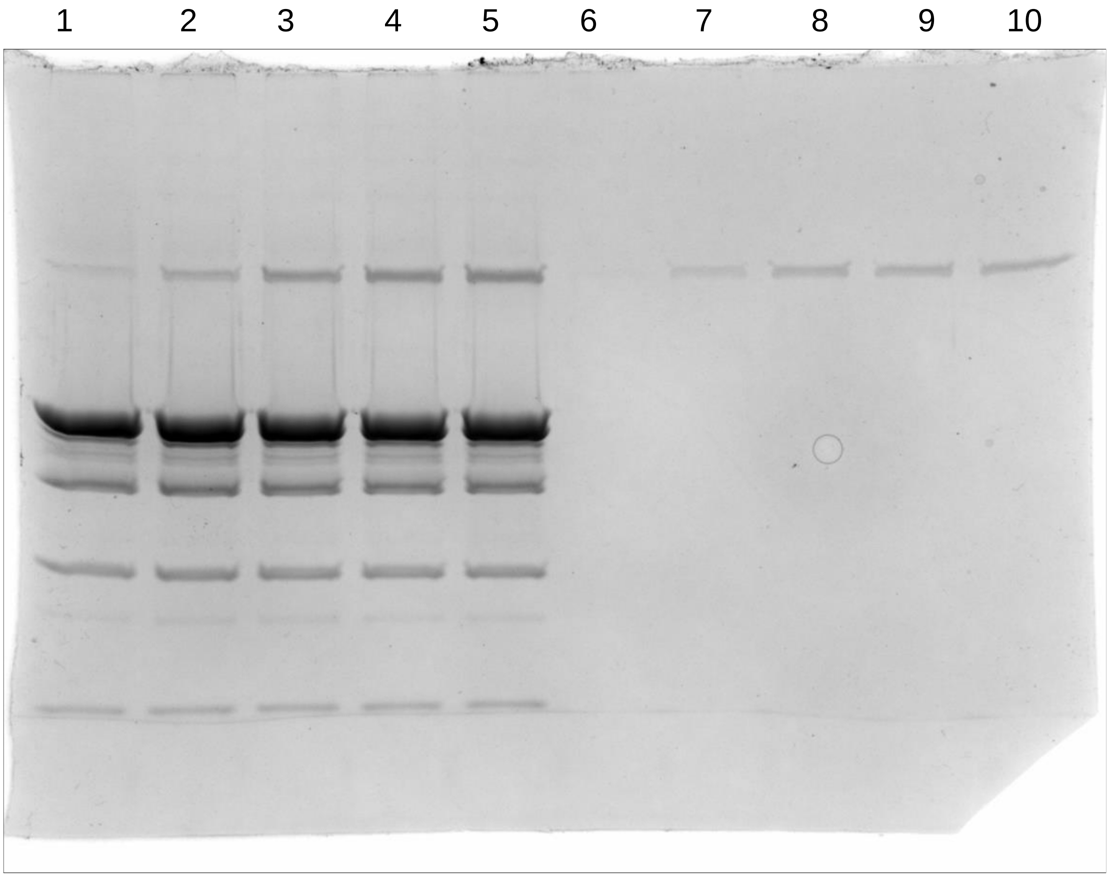

1. 1.5 μM thin filament, 0.25 μM Lmod2[1-524]
2. 1.5 μM thin filament, 0.5 μM Lmod2[1-524]
3. 1.5 μM thin filament, 1 μM Lmod2[1-524]
4. 1.5 μM thin filament, 1.5 μM Lmod2[1-524]
5. 1.5 μM thin filament, 2 μM Lmod2[1-524]
6. 0.25 μM Lmod2[1-524]
7. 0.5 μM Lmod2[1-524]
8. 1 μM Lmod2[1-524]
9. 1.5 μM Lmod2[1-524]
10. 2 μM Lmod2[1-524]

Image captured using Molecular Imager ChemiDoc XRS$^+$ (Bio-Rad)

Data used to make Figure S1B

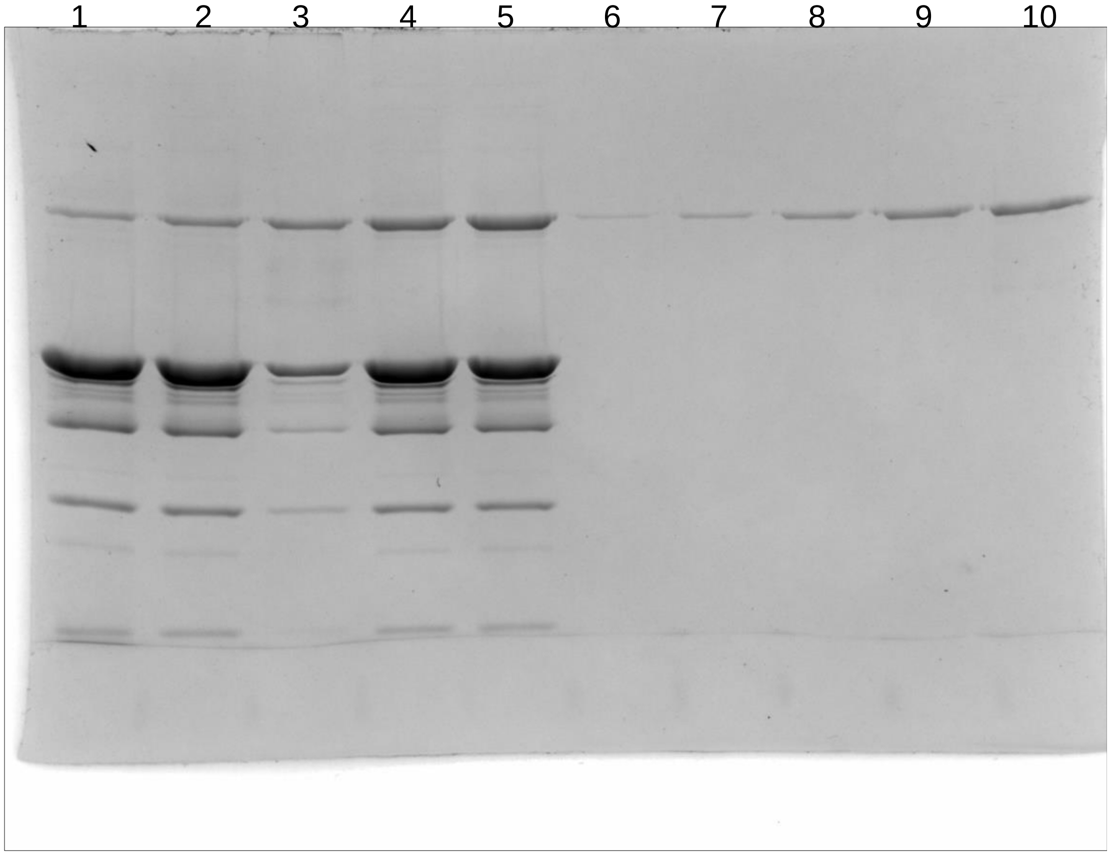

1. 1.5 μM thin filament, 0.25 μM Lmod2
2. 1.5 μM thin filament, 0.5 μM Lmod2
3. 1.5 μM thin filament, 1 μM Lmod2
4. 1.5 μM thin filament, 1.5 μM Lmod2
5. 1.5 μM thin filament, 2 μM Lmod2
6. 0.25 μM Lmod2
7. 0.5 μM Lmod2
8. 1 μM Lmod2
9. 1.5 μM Lmod2
10. 2 μM Lmod2

Image captured using Molecular Imager ChemiDoc XRS^{+} (Bio-Rad)
