# Supplementary material for: Interaction of cardiac leiomodin with the native cardiac thin filament
Source: PLoS Biol. 2025 Jan 30;23(1):e3003027. doi: 10.1371/journal.pbio.3003027 (PMC11813103; doi:10.1371/journal.pbio.3003027)
Supplement: S1 Table — (DOCX) [file pbio.3003027.s012.docx]

|  | **RMSD (Å)** | |
| --- | --- | --- |
| **WH2 Domain** | **Helical** | **Disordered** |
| **hLmod2** | 0.299 | 3.05 |
| **Spire** | 0.192 | 0.553 |
| **WAVE** | 0.196 | 0.699 |

**S1** **Table.** RMSD values of the backbone atoms of aligned WH2 domains in complex with actin monomers as compared to the α-helical (residues 1-10) and disordered (residues 11-17) regions of WASP WH2 domain (from same PDBs as given in S7B Fig).
