# Supplementary material for: Interaction of cardiac leiomodin with the native cardiac thin filament
Source: PLoS Biol. 2025 Jan 30;23(1):e3003027. doi: 10.1371/journal.pbio.3003027 (PMC11813103; doi:10.1371/journal.pbio.3003027)
Supplement: S2 Table — (DOCX) [file pbio.3003027.s013.docx]

**S2 Table.** Data collection and refinement statistics

| **Data collection and refinement statistics** | Ca^2+^-free (pCa>8) TFs in the presence of Lmod2 | Ca^2+^-bound (pCa 3.5) TFs in the presence of Lmod2 |
| --- | --- | --- |
| **Data collection** |  |  |
| Magnification | 65,000 | 65,000 |
| Defocus range, µm | 0.5 – 3.5 | 0.5 – 3.5 |
| Voltage, kV | 300 | 300 |
| Microscope | Titan Krios | Titan Krios |
| Camera | K3 (super-resolution mode) | K3 (super-resolution mode) |
| Number of frames | 40 | 40 |
| Total electron dose, e^-^/Å^2^ | 34 | 34 |
| Frames used for final reconstruction and dose used for final reconstruction, e^-^/Å^2^ | Determined by Relion MotionCorr internal implementation | Determined by Relion MotionCorr internal implementation |
| Pixel size, Å/px | 0.678 | 0.678 |
| **Particle statistics** |  |  |
| Particles | 25,527 | 39,373 |
| Box size, Å | 440 | 440 |
| Pixel size, Å/px | 1.356 | 1.356 |
| **Resolution FSC 0.143, Å** | 6.2 Å map filtered to 9 Å resolution | 6.1 Å map filtered to 9 Å resolution |
